# Gill-associated bacteria are homogeneously selected in amphibious mangrove crabs to sustain host intertidal adaptation

**DOI:** 10.1186/s40168-023-01629-4

**Published:** 2023-08-24

**Authors:** Marco Fusi, David K. Ngugi, Ramona Marasco, Jenny Marie Booth, Massimiliano Cardinale, Luciano Sacchi, Emanuela Clementi, Xinyuan Yang, Elisa Garuglieri, Stilianos Fodelianakis, Grégoire Michoud, Daniele Daffonchio

**Affiliations:** 1 Red Sea Research Center, Biological and Environmental Sciences and Engineering Division, King Abdullah University of Science and Technology, Thuwal, 23955-6900 Kingdom of Saudi Arabia; 2 Centre for Conservation and Restoration Science, Edinburgh Napier University, Edinburgh, UK; 3 Leibniz Institute DSMZ – German Collection of Microorganisms and Cell Cultures, Inhoffenstrasse 7B, D-38124 Braunschweig, Germany; 4 Institute of Applied Microbiology Research Center for BioSystems, Land Use, and Nutrition (IFZ) Justus-Liebig-University Giessen, D-35392 Giessen, Germany; 5 Department of Biological and Environmental Sciences and Technologies, University of Salento, via Prov.le Lecce-Monteroni, I-73100 Lecce, Italy; 6 Dipartimento di Biologia e Biotecnologie “L. Spallanzani”, Università di Pavia, I-27100 Pavia, Italy

**Keywords:** Bimodal breathing, Symbiosis, Gill system, Microbiome, Terrestrialisation

## Abstract

**Background:**

The transition from water to air is a key event in the evolution of many marine organisms to access new food sources, escape water hypoxia, and exploit the higher and temperature-independent oxygen concentration of air. Despite the importance of microorganisms in host adaptation, their contribution to overcoming the challenges posed by the lifestyle changes from water to land is not well understood. To address this, we examined how microbial association with a key multifunctional organ, the gill, is involved in the intertidal adaptation of fiddler crabs, a dual-breathing organism.

**Results:**

Electron microscopy revealed a rod-shaped bacterial layer tightly connected to the gill lamellae of the five crab species sampled across a latitudinal gradient from the central Red Sea to the southern Indian Ocean. The gill bacterial community diversity assessed with 16S rRNA gene amplicon sequencing was consistently low across crab species, and the same actinobacterial group, namely *Ilumatobacter*, was dominant regardless of the geographic location of the host. Using metagenomics and metatranscriptomics, we detected that these members of actinobacteria are potentially able to convert ammonia to amino acids and may help eliminate toxic sulphur compounds and carbon monoxide to which crabs are constantly exposed.

**Conclusions:**

These results indicate that bacteria selected on gills can play a role in the adaptation of animals in dynamic intertidal ecosystems. Hence, this relationship is likely to be important in the ecological and evolutionary processes of the transition from water to air and deserves further attention, including the ontogenetic onset of this association.

Video Abstract

**Supplementary Information:**

The online version contains supplementary material available at 10.1186/s40168-023-01629-4.

## Background

The transition from water to air, known as terrestrialisation, is an evolutionary process that has recurred independently in many animal groups and has contributed to the diversification of terrestrial life forms that we observe today [1–3]. For example, African and South American lungfishes (subclass Dipnoi) have evolved the ability to survive seasonal desiccation by burrowing into mud and estivating throughout the dry season. Physiological changes allow these animals to slow their metabolism to only 1/60th of their normal metabolic rate and convert protein wastes from ammonia to the less toxic urea [4]. The challenges of terrestrialisation are diverse and affect many aspects of animal physiology and behaviour. Osmoregulation and water balance are critical for homeostasis and regulate muscle function and excretion of nitrogenous waste products to avoid accumulation and toxic effects [5].

Among invertebrates, brachyuran crabs are an interesting model for studying the ongoing process of terrestrialisation [6, 7]. Paleontological and biological evidence from extant forms supports the hypothesis that they are at the beginning of the land invasion. Interestingly, the first fossil record of crabs with a semiterrestrial lifestyle dates back to the Cenozoic period, over 60 million years ago [8, 9]. This, in turn, underscores the evolutionary importance of the range of behavioural, morphological and physiological adaptations, namely a modified capacity to excrete nitrogen [10], avoid water loss and exsiccation [11], and develop new morphological traits and organs for gas exchange [12], that have allowed them to successfully occupy a variety of terrestrial and semiterrestrial environments worldwide [13].

In this context, animal gills represent a key multifunctional organ in the adaptation process and the evolutionary path to land colonisation. The gill is responsible for osmoregulation, pH balance of haemolymph, excretion (e.g. nitrogenous wastes), hormonal regulation, detoxification, immune response, and most importantly, oxygen uptake in water and air [14, 15]. Given the direct contact of gills with the surrounding medium (either air or water), they are constantly exposed to environmental toxins and microorganisms. This contrasts with lungs, which are housed in a closed system. Consequently, the gill respiratory surfaces of many marine crustaceans represent portals for the entry and exchange of chemical compounds and facilitate the interaction of the host with allochthonous microbes from their immediate external environment [15]. In particular, the excretion of ammonia and CO_2_ from gill surface creates an ideal environment for microbial colonisation and development that use these nitrogen and carbon resources [16]. However, the role of the gill microbiome in host adaptation remains unresolved. Recent data revealed the colonisation of the gill surface of the brachyuran crab *Eriocheir sinensi*s by a complex bacterial community distinct from the surrounding sediment community [17]. This supports the theory that the microorganisms on the gill surface are not randomly assembled but have mutual relationships with the host and possibly specific functional roles [16].

**Fig. 1:**
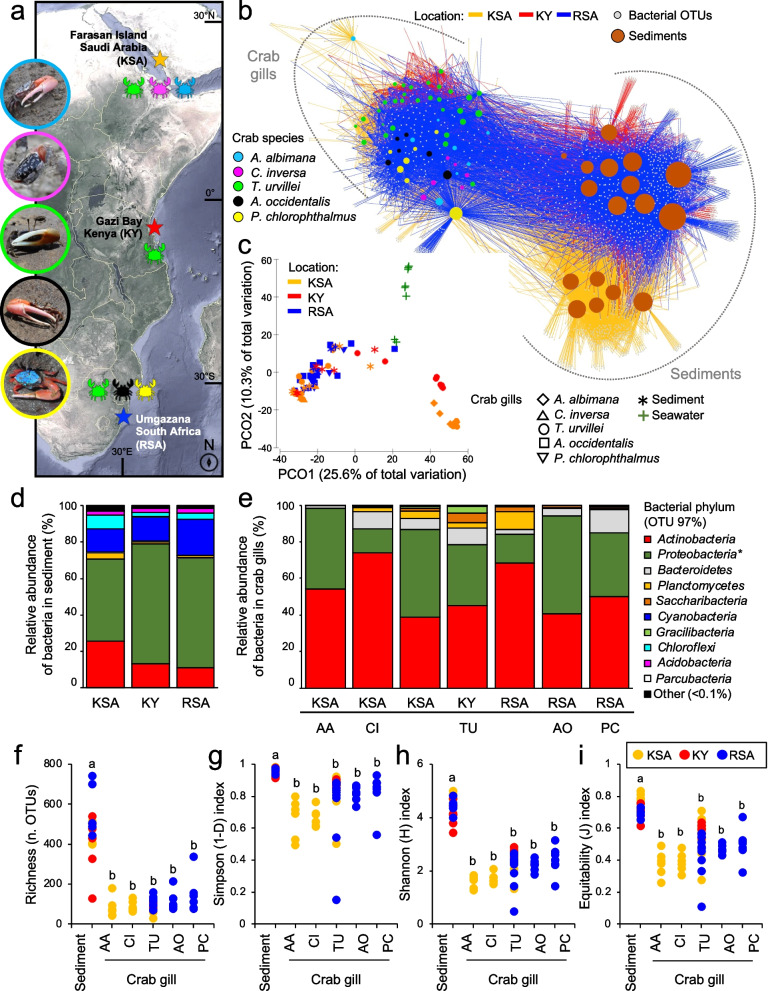
Bacterial microbiome diversity associated with fiddler crab gills. **a** Sampling locations and representative images of crab species sampled (*Austruca albimana* [AA], *Cranuca inversa* [CI], *Tubuca urvillei* [TU], *Austruca occidentalis* [AO], and *Paraleptuca chlorophthalmus* [PC]). **b** Bipartite network shows the relationship of bacterial communities with crab species samples (coloured circles) and sediment (brown circles). Samples clustered based on their shared OTUs (small grey circles). Edges (the lines) indicate if an OTU is present in a certain sample (crabs and sediment circles), and edge colour is associated with the geographical location (blue, Republic of South Africa [ZA]; red, Kenya [KY]; yellow, Kingdom of Saudi Arabia [KSA]). **c** Principal component analysis of gill bacterial microbiome associated with the selected crab species and sediment from the different geographical locations (ZA, KY, and KSA). **d**,** e** Bar plots with the relative abundance of the bacterial phyla retrieved in sediments and gills. The asterisk on “Proteobacteria” indicates that the majority of the OTUs within this phylum belong to *Alphaproteobacteria*. **f**-**i** Alpha diversity indexes of the bacterial community of gill and sediment

Within the context of the holobiont framework, defined as the biological entity involving a host and its associated microbiome [18], we investigated whether the microbiome colonising the gills of geographically isolated fiddler crabs is consistent across diverse species from East Africa to the Red Sea coast (Fig. 1a). The crab species investigated are bimodal breathing (i.e. amphibious, able to breathe in air and water) and undergoing terrestrialisation [19]. Additionally, we examined the potential functional roles of the microbiome, using metagenomics and metatranscriptomics to assess the potential roles of the gill microorganisms in host health and niche adaptation to the harsh and environmentally variable mangrove habitat.

## Results

### The gill microbiome of fiddler crabs differs from the microbiome of sediment and seawater

The gill microbiomes of all the crab species (*Austruca albimana*, *Cranuca inversa*, *Tubuca urvillei*, *Austruca occidentalis* and *Paraleptuca chlorophthalmus*; Fig. 1a) clustered separately from the sediment and seawater microbiomes in terms of bacterial community structure and diversity (Manyglm, Deviance_2,70_=30.88, *p*<0.001; Fig. 1b,c; Table 1; Supplementary Figure S1). This result was highlighted by the bipartite network analysis and the principal component analysis (PCoA) that showed a clear separation of the sediment, seawater and crab gill microbiomes, although many OTUs were shared mainly among sediment and gill and less with the seawater (Fig. 1b,c; Supplementary Figure S2). However, unique OTUs were consistently present in the microbiome of the five crab species sampled in each location that were not found or found less than 500 times in the sediment and seawater microbiome (Fig. 1b,c; Table 2; Supplementary Figure S2). Alpha diversity significantly differed among sediments (ANOVA; Richness, F_10,63_=4.833, *p*<0.05; Simpson F_10,63_=4.653, *p*<0.05; Shannon diversity F_10,63_=5.589, *p*<0.05; Equitability F_10,63_= 4.675, *p*<0.05), and consistent patterns were observed among the crab species along the East African–Saudi Arabian transect (Fig. 1f–i). The significant interaction between crab species and sampling location (Table 1) evidenced a geographically dependent gill microbiome (Supplementary Table S1).

**Table 1:** ANOVA table resulting from the multivariate generalised linear model (manyglm) exploring the different bacterial community composition inferred from the 16S rRNA gene amplicon sequencing among site and species and their interaction. Source are the factors, Res.Df = residual degree of freedom, Df.diff = Degree of freedom, Dev = Deviance, Pr = statistic p

**Source**	**Res.Df**	**Df.diff**	**Dev**	**Pr(>Dev)**
Site	69	3	11961	0.001 ***
Species	64	5	13739	0.001 ***
Site × Species	62	5	2359	0.001 ***

***Indicates statistical significance for the terms analysed

**Table 2:** Relative abundance (%) of OTUs that were consistently detected in all the crabs and rarely found in sediment (i.e. relative abundance <0.01%). Values are reported per species along the three sampling locations (KSA: Saudi Arabia, KY: Kenya, and RSA: South Africa). Taxonomic information of OTUs’ closest relatives obtained from the National Center for Biotechnology Information database is reported as phylum, class, genus/species and accession number. OTUs belonging to the *Ilumatobacter* genus are indicated in bold. Crab species: AA, *A. albimana*; CI, *C. inversa*; TU, *T. urvillei*; AO, *A. occidentalis*; PC, *P. chlorophthalmus*

**OTU_ID**	**KSA**			**KY**	**RSA**			**Phylum**	**Class**	**Closest relative**	**Acc. number**
	**AA**	**CI**	**TU**	**TU**	**PC**	**AO**	**TU**				
**OTU_3**	**67.8**	**43.8**	**15.0**	**13.0**	**33.5**	**24.0**	**20.5**	**Actinobacteria**	***Acidimicrobiia***	***Ilumatobacter* sp.**	**KC817114**
OTU_2	18.9	3.7	8.9	10.6	30.1	26.3	7.6	Proteobacteria	*Phyllobacteriaceae*	*Mesorhizobium* sp.	MH339748
OTU_557	0.9	1.3	1.1	2.7	0.2	4.0	0.8	Proteobacteria	*Rhodobacteraceae*	*Rhodobacteraceae*	KC918105
**OTU_74**	**13.4**	**0.2**	**13.3**	**0.1**	**0.1**	**25.5**	**0.1**	**Actinobacteria**	***Acidimicrobiia***	***Ilumatobacter* sp.**	**KC817109**
**OTU_70**	**2.2**	**2.1**	**0.4**	**0.2**	**7.8**	**1.0**	**0.6**	**Actinobacteria**	***Acidimicrobiia***	***Ilumatobacter* sp.**	**KC817109**
**OTU_120**	**6.6**	**8.0**	**2.7**	**5.7**	**5.9**	**0.0**	**3.7**	**Actinobacteria**	***Acidimicrobiia***	***Ilumatobacter* sp.**	**KC817109**
OTU_14	2.2	1.2	3.5	2.8	7.1	4.1	2.1	Proteobacteria	*Alphaproteobacteria*	*Rhodobium* sp.	JQ806741
OTU_12	0.1	0.1	0.3	0.1	1.8	6.3	0.2	Proteobacteria	*Geminicoccaceae*	*Candidatus* Combothrix	AY590699
**OTU_6**	**2.9**	**0.0**	**2.7**	**1.1**	**19.3**	**1.0**	**1.4**	**Actinobacteria**	***Acidimicrobiia***	***Ilumatobacter* sp.**	**KC018121**
**OTU_5**	**1.6**	**0.0**	**3.1**	**1.9**	**0.6**	**7.4**	**34.0**	**Actinobacteria**	***Acidimicrobiia***	***Ilumatobacter* sp.**	**KC018121**
OTU_16	0.1	1.6	2.9	0.2	0.1	2.6	0.4	Actinobacteria	*Actinobacteria*	*Propionibacteriaceae*	MK947033
OTU_964	0.0	0.1	0.1	0.0	0.4	0.1	0.0	Chloroflexi	*Caldilineae*	*Litorilinea aerophila*	VIGC01000061
OTU_2132	0.2	0.0	0.2	0.0	0.0	0.3	0.0	Proteobacteria	*Alphaproteobacteria*	*Porphyrobacter sanguineus*	FRDF01000003
OTU_4	0.0	0.0	6.2	0.0	0.0	9.9	1.1	Proteobacteria	*Gammaproteobacteria*	*Cardiobacterium hominis*	JQ216520
OTU_15	0.0	0.0	0.4	0.5	5.8	0.4	0.1	Bacteroidetes	*Flavobacteriia*	*Vicingus serpentipes*	VOOS01000003
OTU_13	3.1	2.6	0.0	1.1	4.8	0.0	2.0	Actinobacteria	*Intrasporangiaceae*	*Terrabacter terrigena*	MT415115
OTU_18	0.0	0.0	0.0	4.6	0.0	4.0	2.4	Bacteroidetes	*Weeksellaceae*	*Bergeyella* sp.	KC203058
OTU_21	0.3	0.4	1.3	1.7	0.3	0.9	0.7	Actinobacteria	*Propionibacteriaceae*	*Naumannella*	MH127702

Taxa dominating bacterial communities (>0.1% of relative abundance) associated with the sediment and crabs’ gill included Actinobacteria (on average 15% and 55%, respectively) and Proteobacteria (on average 61% and 33.9%, respectively); these two phyla together with Bacteroidetes (in average 6.5%) were the most abundant, and *Ilumatobacter* (within Actinobacteria) accounted for up to 50% in the gill microbiome (Table 2). Notably, OTUs belonging to the genus *Ilumatobacter* were almost absent (range 0.1%–0.8% relative abundance) in aquatic crabs, such as *Thalamita crenata* collected from the Red Sea (Supplementary Figure S3). The more heterogenous sediment and seawater were also inhabited by Cyanobacteria, Chloroflexi and Acidobacteria that we found only in low abundance in the gills (Fig. 1d,e). Sloan’s Neutral Community Model confirmed that sediment microbiomes in South Africa (RSA) and Saudi Arabia (KSA) were well-homogenised (*m*=0.849, R^2^=0.667 and *m*=0.756, R^2^=0.674, respectively), as was Kenyan sediment microbiomes (KY) but to a lesser extent (*m*=0.211, R^2^=0.58). At the same time, this model discarded a source-sink hypothesis from sediments to gills (R^2^ consistently negative, irrespective of crab species), suggesting that the sediment microbiome does not represent a pool from which bacteria randomly spread into the gills.

### Phyloscore analysis reveals homogenous bacterial selection on crab gills

**Fig. 2:**
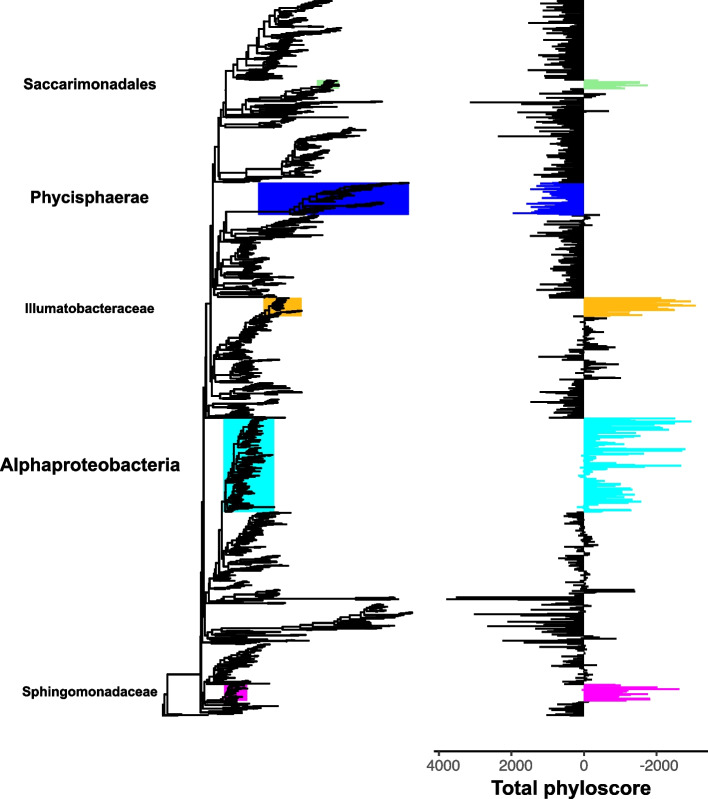
Identification of phylogenetic clades with distinct phylogenetic turnover patterns compared to the rest of the microbiome across all samples. The phylogenetic tree concerns all OTUs from all samples. Clades containing more than 15 OTUs are colour-coded on the phylogenetic tree, and the consensus taxonomy is given for each clade on the left with font size proportional to taxonomic breadth. The total phyloscore of each clade (i.e. the sums of the phyloscores across community pairs) is shown to the right as bars with colours matching the clades’ colours. Negative phyloscores indicate clades that contain OTUs whose closest relatives (across samples) reside at shorter phylogenetic distances than those expected by chance, and vice versa. Thus, negative phyloscores indicate clades having lower-than-expected phylogenetic turnover (i.e. clades with a niche across all crab gills) and vice versa

We found five phylogenetic clades with distinct phylogenetic turnover patterns compared to the rest of the microbiome (Fig. 2; Supplementary Figure S4), i.e. having significantly different total phyloscores compared to the rest of the microbiome (contrast tests, 10^−8^>*p*>10^−57^). Four out of these clades had a lower-than-expected phylogenetic turnover, indicating that their members can be found more frequently than expected by chance across all gill samples. Interestingly, a clade of 29 OTUs affiliated with *Ilumatobacteraceae* had the lowest total phyloscores on average. The other three clades with low phylogenetic turnover were affiliated with *Alphaproteobacteria* (149 OTUs)*, Sphingomonadaceae* (26 OTUs), and *Saccharimonadales* (14 OTUs). However, one clade (51 OTUs) had higher-than-expected phylogenetic turnover, indicating that it is preferentially found in a subset of samples, and was taxonomically affiliated to *Phycisphaerae* belonging to the phylum Planctomycetes particularly abundant in the crab species *T. urvillei*.

### Bacteria are tightly attached to the gill lamellae

**Fig. 3:**
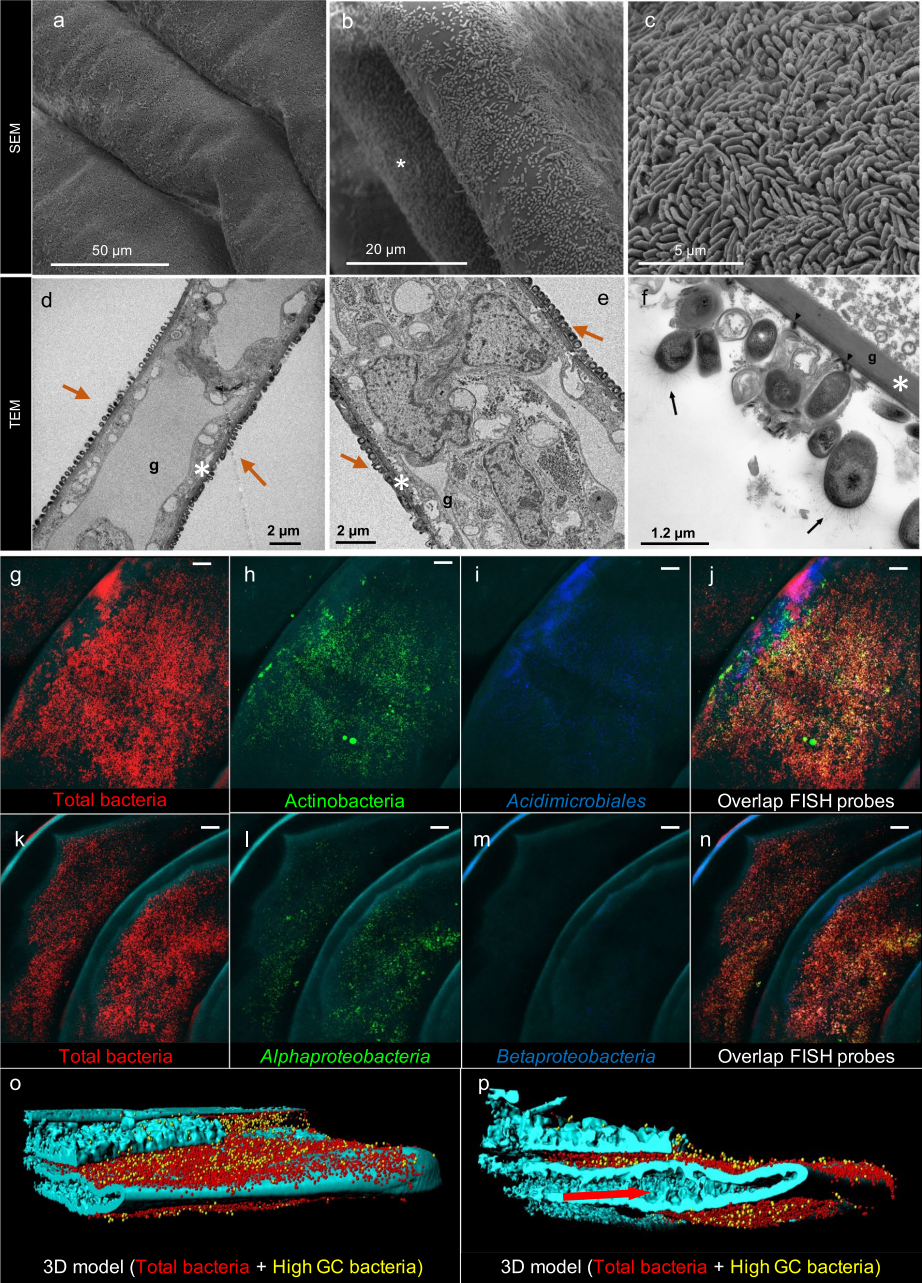
Distribution of microbial communities over the gill lamellae of fiddler crabs. **a**–**c** Scanning electron microscope (SEM) imaging of a representative gill of the fiddler crabs *Tubuca urvillei*. Representative SEM images of the other crab species gills are provided in Supplementary Figure S5. **a** Magnification of the gill lamellae. **b** Detail of the bacterial layer covering the lamellae of the gill of each species investigated. Asterisks indicate the gill lamellae. **c** Rod-like bacteria covering the entire lamellae of the gills. **d–f** Transmission electron microscope imaging of fiddler crab gill (indicated by a “g” letter) of *Austruca albimana*: white asterisks show the gill cuticles, while orange arrows the bacterial layer. **d** Section of the gill that shows both side of the gill lamellae covered by bacteria. **e** Magnification of the bacterial layer. **f** Gill lamellae surface (indicated by a g letter); arrowheads: electro-dense area where bacteria are attached to the gill; black arrows: bacterial pili. **g**–**p** Localisation of bacteria in crab gill by confocal laser scanning microscopy (CLSM) of fluorescence in situ hybridisation (FISH)-stained gill lamellae of *T. urvillei* (**g–j**) and *A. albimana* (**k**–**n**) with the different probes (see supplementary figure S5 for the bright field and FISH negative control). **o**,** p** IMARIS 3D-structure reconstruction of the gill lamellae and the bacterial layer (frontal and lateral view) of *T. urvillei*. Note the absence of signal inside the gill lamellae that support the evidence that bacteria live on the surface of the lamellae without entering them. Red arrow indicates the heterogeneous inner morphology of the gill lamellae. “High CG bacteria” indicates cells with high guanine-cytosine content typical of Actinobacteria. The images are meant to be typical of the range of observations made

In all crab species examined, SEM imaging revealed the consistent presence of a bacterial layer coating the gills and extending across the ridges of each lamella (Fig. 3a; Supplementary Figure S5), confirming recent results obtained by Garuglieri and collaborators [16]. Further magnification showed a homogeneous layer of rod-shaped bacteria covering the gill surface of all collected specimens (Fig. 3b,c; Supplementary Figure S5), which TEM confirmed (Fig. 3d,e). TEM imaging also revealed a tight association between bacteria and the outer edge of the lamellae through the formation of connections (i.e. large electro-dense areas indicated by the arrowhead in Fig. 3f) that anchor the bacterial cells to the surface of the gill lamellae. Where the electro-dense filaments contact the gills, the lamellar surface is distorted (Fig. 3f and [16]), indicating that these connections are strong. In addition, the attached bacterial cells had pili and nanowire structures and were embedded in an extracellular matrix (Fig. 3f).

Fluorescence in situ hybridisation (FISH; see probes in Supplementary Table S2) performed on *A. albimana* confirmed the results obtained by electron microscopy and molecular 16S rRNA gene metabarcoding, revealing a bacterial layer on the upper surface of the gill lamellae (Fig. 3g–p). 3D-reconstruction of gill FISH imaging showed no bacterial cells within the lamella vessels, which exchange gases from/to haemolymph and excrete catabolites [20] (Fig. 3o,p; Supplementary Video 1). The negative FISH control showed no probe-transmitted signals in the samples stained with NONEUB (Supplementary Figure S6). Of the total bacterial cells, most belonged to Actinobacteria (order *Acidimicrobiales*) and *Alphaproteobacteria*.

### The gill microbiome is functionally distinct from the sediment microbiome

To gain insight into the process that shapes the gill microbiome of crabs, we conducted a gene-centric metagenomic survey of the resident prokaryotic communities on the gills of four geographically isolated fiddler crab species (*T. urvillei*, *C. inversa*, *A. albimana* and *P. chlorophthalmus*; *n* = 2–3 individuals per site) compared to microbial communities in the adjacent sediments (*n* = 8; Supplementary Table S3). A total of 208 gigabase pairs were recovered from high-quality paired-end reads (Supplementary Table S3). Taxonomic assignment of putative protein-coding genes predicted from independent metagenomic assemblies (Supplementary Figure S7) revealed the predominance of bacterial genes in the coding sequence space, with a significant enrichment (unpaired *t*-test, *p*<0.0001) of genes from the phylum Actinobacteria in the gill microbiome compared to the sediments (41% ± 3.7% vs 8.7% ± 2.2%). Importantly, 7% to 35% (mean ± SD 16% ± 7%) of the bacterial genes in the gill microbiome were from the genus *Ilumatobacter*, but only ~1% to 6% in the sediment microbiome. In contrast, the sediment microbiome was significantly enriched (unpaired *t*-test, *p*=0.0045) with genes from the phylum Bacteroidetes (21% ± 3% vs 8.2% ± 2.4%) and Cyanobacteria (8.5% ± 2.8% vs ~1%). Similar trends in community composition were evident in unamplified 16S rRNA gene sequences obtained from the same metagenomes (Supplementary Figure S8). Overall, these findings support those based on 16S rRNA gene amplicon data (see Fig. 1), showing the predominance of Actinobacteria and Proteobacteria in the crab gill microbiome.

**Fig. 4:**
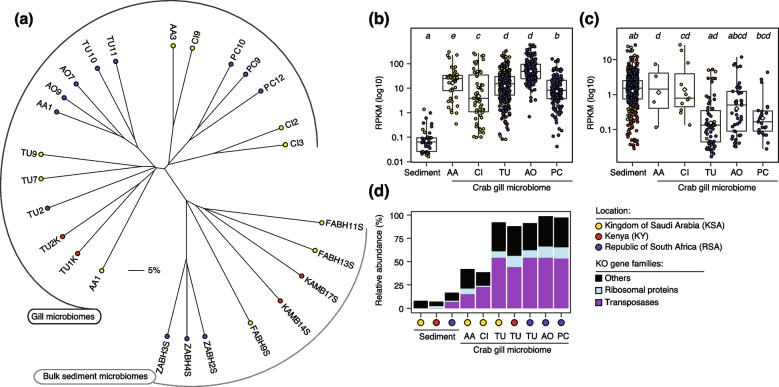
Metagenomics distinguishes the functional repertoire of the fiddler crab gill microbiome from the neighbouring sediment microbiome. **a** Grouping of gill and sediment microbiome samples (2–3 independent replicates per species/site) based on the Bray-Curtis dissimilarity index calculated using abundance/coverage of a random set of 100,000 genes in the annotated gene catalogue of both microbiomes. Crab species and site samples are colour-coded by location (same as in Fig. 1), and crab names are labelled according to crab species names: *T. urvillei* (TU), *C. inversa* (CI), *A. albimana* (AA), *A. occidentalis* (AO), and *P. chlorophthalmus* (PC); see Supplementary Table S1 for more details. **b**, **c** The abundance of the fifty most highly represented functions based on random forest predictions in gill (**b**) and sediment (**c**) microbiomes (2–3 replicates per sample). The plots represent independent gene sets that may have a few copies with low coverage in the gill or sediment microbiome, explaining the additional *x*-axis labels despite these being abundant in either the gill or sediment microbiomes. Boxplots show median coverage as the middle horizontal line and interquartile ranges as boxes (whiskers extend no further than 1.5× the interquartile range). Circular symbols reveal the diversity of enriched genes, with colours reflecting the location of the sample. Mean values are shown as white coloured diamonds. Different lowercase letters at the top of each boxplot denote significance differences based on the two-tailed unpaired Wilcoxon test (*p*<0.05). RPKM, reads per kilobase per million mapped reads. **d** Bar graphs show the high proportion of genes encoding transposases among the fifty most enriched annotated KOs in the crab gill microbiomes relative to the sediment microbiome

A nonredundant gene catalogue of the crab gill microbiome was constructed by clustering ~1.8 million protein-coding genes from all samples at 95% global sequence identity over 80% of the shorter gene length (details in “Methods”). Functional annotation showed that half of the predicted 1.64 million nonredundant genes encoded putative functions in the KEGG database (737,124 genes). The majority (554,439 nonredundant genes) were taxonomically assigned to prokaryotes (95% Bacteria; ~2% Archaea; Supplementary Figure S9). Next, we inferred the coverage of these catalogued crab gill and sediment microbiome genes based on mapping them against individual metagenomes. To account for variations in sequencing depth, subsequent analyses focussed on a random set of 100,000 nonredundant genes (details in “Methods”) with KEGG Orthology (KO) identifiers assigned to prokaryotes (bacteria and archaea; Supplementary Figure S9) and a normalised gene abundance cut-off of 0.001 RPKM (reads per kilobase per million mapped reads). Hierarchical clustering of samples based on the Bray-Curtis dissimilarity index showed a clear separation of gill microbiome samples from sediment samples (Fig. 4a), albeit with no clear separation of gill microbiome samples by species (2–3 replicates per species and site). Therefore, we investigated whether certain functions were prominent in the gill microbiome relative to the sediment microbiome by focusing on the whole set of KOs with the highest coverage based on random forest predictions (details in “Methods”). The top fifty KOs that distinguished gill microbiome functions from sediment microbiome functions also had significantly high coverage in the crab gill than in the sediment samples (two-tailed unpaired Wilcoxon test, *p* < 0.05; Fig. 4b, c). For example, normalised mean coverage (2–3 replicates per sample) was tenfold higher in the gills than in the sediment samples (29 ± 3 vs 2.2 ± 0.4 RPKM; Fig. 4c). Remarkably, 15–54% (27 unique genes) of these fifty KO families enriched in the gill microbiome encoded transposases (Fig. 4d). In contrast, a much greater KO diversity (39 unique genes) was enriched in sediment microbiomes, comprising functions such as urea mineralisation and carbon monoxide oxidation. Independent of the microbiome, the fifty most enriched functions were found to be mainly from members of the phylum Proteobacteria (72%–74%); however, a slightly higher proportion of Actinobacteria was also found in the fifty-topmost abundant gill microbiome genes (22%) than in the sediment microbiome (~17%).

### The gill microbiome encodes key metabolic functions relevant to host physiology

**Fig. 5:**
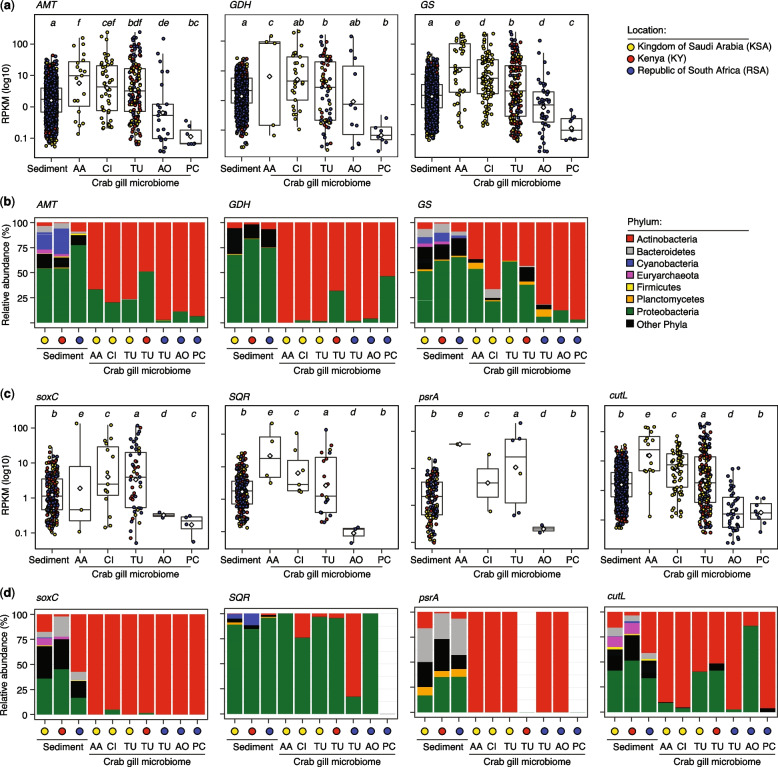
Abundance and taxonomic origin of key enzymes encoded by the gill microbiome with relevance to host physiology. **a**,** c**, **d** Boxplots show the normalised abundance of seven different metabolic genes in reads per kilobase per million mapped reads (RPKM) in the microbiome associated with sediment and gills of different crab species. The circular symbols show the mean RPKM value (log-scaled) for each predicted unique gene copy (per enzyme; *n* = 2–3 replicates) and are coloured according to the sampling location. The mean RPKM value of all genes is shown as a horizontal bar. Different lowercase letters on the *x*-axis indicate microbiomes with significantly different mean (RPKM) abundances as determined by a two-tailed, unpaired Wilcoxon test (*p* < 0.05). In all cases, gene family diversity is higher in the sediment microbiome than in the gill microbiome of all crab species. **b**,** e**, **f** Bar graphs show the predicted taxonomic origin (at the phylum level) of genes encoding enzymes in the nitrogen (**b**), sulphur (**e**), and carbon (**f**) cycles in the gill microbiome relative to the sediment microbiome. The percentages in the bar graphs show the relative abundance of each phylum based on the aggregated RPKM of all genes shown in the individual boxplots. Gene abbreviations (and corresponding KO identifiers): *AMT*, ammonia transporter (K03320); *GDH*, glutamate dehydrogenase (K15371); *GS*, glutamine synthetase (K01915); *soxC*, sulfite oxidase (K00387); *SQR*, sulphide:quinone oxidoreductase (K17218); *psrA*, polysulfide reductase subunit A (K08352); and *cutL*, aerobic carbon monoxide dehydrogenase, large subunit (K03520). Abbreviations of crab species: AA, *A. albimana*; CI, *C. inversa*; TU, *T. urvillei*; AO, *A. occidentalis*; and PC, *P. chlorophthalmus*. RPKM, reads per kilobase per million mapped reads

The gill is an essential organ for respiration and waste exchange in crabs, which is also exposed to environmental stressors, such as toxic gases (e.g. hydrogen sulphide, methane, and carbon monoxide) released in the predominantly anoxic sediments [21–23]. Accordingly, we theorised that the gill microbiome has the genetic potential for the assimilation of gill excretory products such as ammonia waste [14] and conversion into useful metabolites (e.g. amino acids) for the holobiont. To this end, we analysed the relative abundance of key enzymes in the corresponding microbially catalysed metabolic pathways and their putative taxonomic origin (Fig. 5; Supplementary Figure S10). The results showed that the copies of these key enzymes were less diverse and taxonomically restricted in the gill microbiome of different crab species than in the neighbouring sediments (Fig. 5).

The abundance of these genes based on metagenomic mapping coverage showed that those involved in the exchange of ammonia (via the ammonia transporter AMT) and its assimilation to amino acids via pathways catalysed by NADPH-dependent glutamate dehydrogenase (GDH) or ATP-dependent glutamine synthetase (GS) were significantly enriched (Kruskal-Wallis test *χ*^*2*^ = 34–125, df = 5, *p*<0.0001) in the gill microbiome of three crab species (*A. albimana*, *C. inversa*, and *T. urvillei*) compared with the sediment microbiome (Fig. 5a). Notably, bacterial and archaeal ammonia monooxygenase genes (*amoA*, *amoB*, and *amoC*) were absent from the gill microbiome. However, only one *Nitrosopumilus*-like gene was found in the sediment samples, suggesting that no canonical nitrifiers on the gills assimilate ammonia directly excreted by the host to nitrite. Remarkably, up to 50% of these three over-represented gill microbiome nitrogen cycle genes (*AMT*, *GDH*, and *GS*) were assigned to members of the phylum Actinobacteria (Fig. 5b), mainly the genus *Ilumatobacter*, making up between 20 and 60% of the Actinobacteria assignments and roughly 4%–10% of the AMT, GDH, and GS genes.

The gill surface also provides a selective interface between the external seawater and the anoxic mangrove sediments, and the internal gill tissues. Thus, it is an important entry port for the accumulation of toxic substances (e.g. hydrogen sulphide and carbon monoxide) from the coastal sediment’s anoxic (and polluted) environment. Hence, we additionally investigated whether the gill microbiome encodes functions relevant to the detoxification of sulphur compounds (H_2_S) and carbon monoxide (CO), which are likely to be present at higher concentrations in the sediment-seawater interface and in the sediment. For example, coastal marine sediments contain up to 200 µM H2S [24] and 10 µM CO [25] that can impact gill functions and homeostasis; sublethal concentrations as low as 0.3–20 µM inhibit oxygen storage and mitochondrial respiration activity [26]. This implies that the gill tissue, the gill-associated microbiome, or both must be involved in controlling H_2_S and CO bioavailability to avoid cytotoxicity. Metagenome profiling of relevant detoxifying metabolic pathways revealed a lower diversity in the gill microbiome than in the sediment microbiome, along with an enrichment of genes encoding sulphide-oxidising metabolic pathways in the gill microbiome, including sulphur dioxygenase (SoxC), sulphide-quinone oxidoreductase (SQR), and persulfide dioxygenase (PrsA) (Fig. 5c). These three sulphur cycling systems likely play a critical role in maintaining nontoxic concentrations of H_2_S within the gills [27]. Similarly, our results showed that the gill microbiome is enriched in the *cutL* gene, encoding aerobic carbon monoxide dehydrogenase for oxidising CO to nontoxic intermediates (Fig. 5d). As it is observed for the nitrogen cycling pathways enriched in the gill microbiome, many of the genes involved in the H_2_S and CO detoxifying pathways were associated with members of the phylum Actinobacteria, whereas diverse organisms encoded these functions in the sediment microbiome (Fig. 5).

### Actinobacteria-derived detoxification pathways are expressed in the gill tissues of wild crabs

**Fig. 6:**
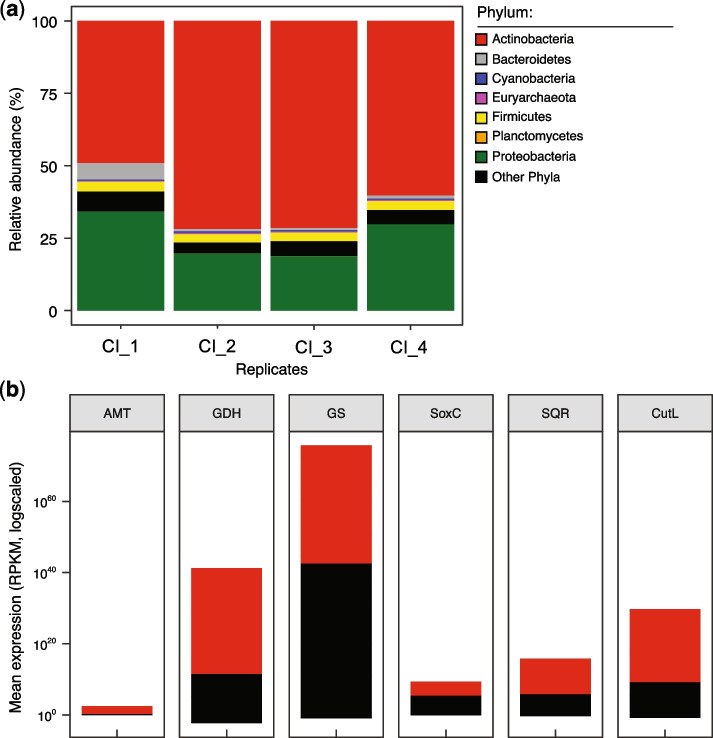
Expression of ammonia, sulphide, and carbon monoxide detoxification pathways in the gills of wild crabs. **a** Taxonomic assignment of bacteria transcripts assembled from gill tissue metatranscriptomes (CI_1–CI_4) of the fiddler crab *C. inversa* collected from the Red Sea coast near KAUST (related to KSA samples). Bar plots show the relative proportion of total transcripts assigned to prokaryotes (details in the “Methods”) in four independent animals. **b** Normalised mean expression (as RPKM values log-scaled) of key enzymes involved in nitrogen (AMT, GDH, and GS), sulphur (soxC and SQR), and carbon (cutL) metabolism, most of which are derived from Actinobacteria in the case of *C. inversa.* Gene abbreviations (and corresponding KO identifiers): *AMT*, ammonia transporter (K03320); *GDH*, glutamate dehydrogenase (K15371); *GS*, glutamine synthetase (K01915); *soxC*, sulphite oxidase (K00387); *SQR*, sulphide:quinone oxidoreductase (K17218); and *cutL*, aerobic carbon monoxide dehydrogenase, large subunit (K03520). No *psrA*, polysulfide reductase subunit A (K08352), was detected. RPKM (reads per kilobase per million mapped reads) denotes normalised expression levels*.* The red stack of the bar plot represents Actinobacteria and the black stack of all the other bacterial phyla

We used metatranscriptomics (Supplementary Table S6) to investigate whether the metabolic pathways retrieved from microbial genomes are expressed in crab gills. We examined the expression of the corresponding genes, namely *AMT, GDH*, *GS*, *SQR*, *soxC*, *psrA*, and *cutL*, from reconstructed gill tissue transcripts of *C. inversa* (*n* = 4 independent animals). We found that the transcripts of these genes were linked to members of the Actinobacteria phylum and accounted for a substantial proportion (~50%–72%) of the actively transcribed prokaryote-derived genes in the gill microbiome (Fig. 6a). Actinobacterial genes for the transport/exchange of ammonia (*AMT*) and conversion to different amino acids (*GDH* and *GS*) were highly expressed in *C. inversa* (Fig. 6b). The same was observed for genes encoding enzymes involved in the oxidation of carbon monoxide (cutL) and hydrogen sulphide (soxC and SQR; Fig. 6b). No transcripts of *psrA* were found. Importantly, most of the transcripts for *GDH* (3–32%), *GS* (2%–5%), and *cutL* (61%–95%) were from the genus *Ilumatobacter*, suggesting the potential role of this actinobacterial group in nitrogen recycling and detoxification of carbon monoxide. Overall, the results highlight the importance of bacteria in C, N, and S cycling and homeostasis of *C. inversa* gills.

To support our hypothesis that bacteria may be enriched for ammonia recycling, we measured the ammonia concentration in the gills of semiterrestrial fiddler crab *C. inversa* and compared it to that produced by the gills of sympatric aquatic crab *T. crenata* (see Supplementary Method S1 for ammonia quantification). The result showed that the fiddler crab has a significantly higher ammonia level than the aquatic crab (Supplementary Figure S3b). Notably, the higher level of ammonia in *C. inversa* is associated with the higher presence of *Ilumatobacter sp.* in the gills, which is almost absent (0.1%–0.8%) in the aquatic species where the level of ammonia is significantly lower (Supplementary Figure S3). These results confirm that *Ilumatobacter*, having an active pathway for ammonia recycling metabolism, occupy the ammonia-rich niche of intertidal crabs such as *C. inversa*.

## Discussion and conclusions

The transition from sea to land in dynamic coastal habitats presents a unique challenge for any organism [28] due to broad gradients of oxygen, nutrients, salinity, and temperature that significantly affect their physiology and ecology [29]. This is the case for fiddler crabs inhabiting the intertidal mangrove habitat [30]. Fiddler crabs are semiterrestrial organisms included in a pantropical taxonomic group that has successfully colonised mangrove forests [31], where they act as ecosystem engineers and play a vital role in mediating the geochemistry and shaping the ecology of mangrove sediments [32–35]. Coping with environmental gradients inherent in these habitats necessitates the development of an amphibious lifestyle to thrive in the intertidal environment, including physiological [19], behavioural [36, 37], and anatomical adaptations [38]. However, the role of host-associated microorganisms in the evolution of the host toward terrestrial environments and the resulting adaptation to the challenging intertidal mangrove habitat has remained unresolved.

Previous studies have shown that crab gills can harbour parasites or commensal organisms [39], but also a consistent and close association with bacteria has been documented in the Chinese Mitten crab [17] and recently in mangrove crabs [16]. The present study demonstrated that five fiddler crab species consistently possess a dense layer of bacteria covering the gill lamellae, regardless of geographic location. Our findings add to the growing literature on the central role of gill-associated bacteria in aquatic organisms [40, 41] that bacteria on the gill of mangrove crabs may contribute to ammonium waste removal, nitrogen recycling and protection from sulphide and other toxic gases during respiration.

The gills of bimodal amphibious animals, such as fiddler crabs, are an essential organ for respiration, excretion, acid/base regulation, and protection from xenobiotics [14]. Their role in oxygen uptake is at odds with our discovery that crab gills harbour a thick layer of bacteria covering such a functionally important organ for respiration. The presence of aerobic bacteria on the gills [16] means that they compete with the host for the oxygen that diffuses through the gills and consequently has potentially deleterious effects on the host physiology. On the other hand, the transition to land and the associated exposure to higher oxygen concentration may represent an oxidative stress that the bacteria can buffer by scavenging the oxygen [42]. Images of the gill tissue showed an intimate association of bacteria with the surface of the gill lamellae, including the formation of electro-dense structures (Fig. 3a–f) embedded in the chitinous surface of the gills and, in some cases, even altering the morphology of the surface itself. These electron-dense structures could be nanowires for electron exchange [43] and/or nutrient and signalling molecules [44].

Marker gene survey and whole-genome sequencing revealed two main bacterial taxa associated with gill tissues independent of the geographic origin of the host: members of the classes *Acidimicrobia* (Actinobacteria), mainly the genus *Ilumatobacter*, and *Alphaproteobacteria* (Proteobacteria), specifically *Rhizobiales* and *Sphingomonadales*. In particular, the family *Ilumatobacteraceae* showed significantly low phylogenetic turnover across all gill samples, suggesting a common niche for this clade in the gills of fiddler crabs. These results confirm evidence from independent reports from a previous study of the Chinese Mitten crab *Eriocheir sinensis* [17]. Overall, the results suggest host independence and a highly selective bacteria acquisition, as the gill microbiome composition is not significantly affected by the surrounding sediment microbiome by means of random dispersal and establishment or host geography. In turn, the presence of the same major taxa in geographically isolated hosts implies that selection is both at the species and functional levels. As found in some fishes [45], lower taxonomical and functional diversity on the gill indicates the high selectivity of the gill environment on the resident microbiome, with fewer adapted bacterial species than in the surrounding environment. This is reinforced by the particularly high abundance of transposase in the gill microbiome of the fiddler crabs, which is also observed in endosymbiotic bacteria like those found in the gutless marine worm *Olavius algarvensis* [46].

Gene-centric analysis of the gill metagenomic and metatranscriptomic data (Figs. 5, 6) revealed the capacity of Actinobacteria to exploit nitrogenous excretory compounds of the host, such as ammonium, reflected in the prevalence of the nitrogen cycling pathways carried mostly by *Ilumatobacter* in the gill microbiome of different fiddler crab species (Fig. 5). Given the intimate embedment of Actinobacteria-like microbes in the gill tissues, this suggests the potential role of the resident *Ilumatobacter* species in gill homeostasis, including the removal of excretory ammonia—a potential toxin that causes tissue damage, which can be converted to essential amino acids for the holobiont. In ammoniotelic aquatic species, such as fishes and molluscs, ammonia is excreted by the gill [14] and represents a readily available source of nitrogen that bacteria can use to produce amino acids. Significantly, members of *Ilumatobacter* are also associated with marine sponges [47], and those we found on the crab gills are closely related to cultured representatives (*I. fluminis*, *I. coccineum*, and *I. nomiense*) that possess the potential to synthesise several amino acids and essential cofactors [48, 49].

In most aquatic animals, ammonia is excreted through the branchial epithelium as NH_3_ via a favourable blood-water diffusion gradient [50]. However, in the process of terrestrialisation, ammonia excretion may be curtailed during emersion because the branchial and cutaneous surfaces are no longer surrounded by water, which in turn leads to high ammonium accumulation in the unstirred water layer of the branchial epithelium [5], as observed in *C. inversa* compared to the aquatic crab *T. crenata* (Supplementary Figure S3). This is postulated to be the case in amphibious species, such as the fiddler crabs studied here. Decapod crustaceans exhibit a variety of mechanisms for excreting ammonia, for instance, via NH_3_ gas diffusion or by active transport of protonated NH_4_^+^, depending on environmental conditions and the taxa considered [51]. Intertidal crabs have a clear continuum of increased terrestriality accompanied by controlled and active excretion with a reduced water loss [13].

Of ecological relevance, fiddler crabs are active at low tide and therefore breathe air. During submergence, they usually retreat to their burrows, which lowers their metabolic rate [19]. Increased metabolic activity in the air may cause the animal difficulty excreting ammonia. Bacteria can resolve this problem by scavenging ammonia excreted through the gill tissue, thus restoring a favourable diffusion boundary for the detoxification of ammonia. Indeed, metagenomic results show an overrepresentation of microbially associated pathways for ammonium uptake (via AMT) and assimilation to amino acids (via NADPH-dependent glutamate dehydrogenase or the ATP-dependent glutamine synthetase) in the gill microbiome. Also, AMT may create resistance to antibiotics or xenobiotics [52–56].

Interestingly, unlike in other aquatic animals [45], no canonical nitrifiers capable of oxidising ammonia excreted by the host to nitrite were detected in the gill microbiome. We suggest that the absence of this metabolic group in the crab gill microbiome is likely due to (1) the strict selection of microbial partners that assimilate ammonia to essential amino acids for the host (i.e. *Ilumatobacter* members)—rather than to the toxic ammonia oxidation by-product nitrite and (2) the inhibitory effect of high ammonia in the gill environment (around 12–26 µmol g^–1^ fresh weight h^–1^ is 

actively excreted by the shore crab *Carcinus maenas* [14, 51]) or sulphide concentrations in mangrove sediments (up to 200 µM H2S [24]) on nitrifiers in general [57]. There is evidence that lucinid bivalves living in mangrove ecosystems use symbiotic bacteria to oxidise H_2_S and avoid the harmful effects of this gas [58]. We postulate that the bacteria on the gills of fiddler crabs perform a similar protective function by removing sulphur compounds (such as H_2_S) and carbon monoxide (CO), often present when the animals enter the sediment-water interface. This is supported by the presence of genes encoding enzymes that catalyse the respective detoxification pathways in the gill microbiome, such as sulphur dioxygenase (SoxC), sulphide-quinone oxidoreductase (SQR), and persulphate dioxygenase (PrsA) for H_2_S and aerobic carbon monoxide dehydrogenase (cutL) for CO, which are deduced to come from the gill-associated bacterium *Ilumatobacter*. Eliminating hydrogen sulphide and carbon monoxide allows the maintenance of nontoxic concentrations of these gases in the gills.

Our study shows that the ensemble of functions encoded in the gill microbiome of fiddler crabs might provide strategies for host adaptation towards life on land and enable coping with challenging and dynamic intertidal life. The discovery of a novel and gill-specific microbiome widespread in various geographically separated fiddler crabs implies an operational evolutionary process that selects for actinobacteria of the genus *Ilumatobacter*. Actinobacteria are metabolically versatile and, when associated with gills, can provide critical metabolic functions for host adaptation to the dynamic mangrove ecosystem. Gill bacteria encode functions and provide mechanisms that facilitate the adaptation to salt and heat stress, anoxia, and detoxification of gaseous toxins and environmental pollutants. In turn, the combined prevalence of *Ilumatobacter* in the gill microbiome of several fiddler crab species and the demonstrated genetic capabilities provide an ideal model to elucidate the phylogenetic and functional selectivity of the microbiome associated with bimodal mangrove crabs. This also reveals the possible roles of gill bacteria in host adaptation to the physiological challenges the animals encounter in the passage from the sea to the land, providing valuable insights into the mechanisms that are likely to be important in the ecological processes of the transition from water to air. Further investigation will be pivotal to unveil the evolutionary role of this symbiome, including the ontogenetic onset of this bacterial-host association.

## Methods

### Sampling and DNA extraction

Three mangrove forests were chosen for mangrove crab collection (Fig. 1a): Farasan Island (Jazan district, Kingdom of Saudi Arabia; 16° 47′ N, 42° 39′ E), Gazi Bay (Kwale district, Kenya; 4° 22′ S, 39° 30′ E), and Mngazana forest (Eastern Cape province, South Africa; 31° 42′ S, 29° 25′ E). The following specimens were collected: *Tubuca urvillei* (TU - H. Milne Edwards, 1852), *Austruca albimana* (AA - Kossmann, 1877), *Cranuca inversa* (CI - Hoffmann, 1874), *Thalamita crenata* (Rüppell, 1830) from Saudi Arabia; *Tubuca urvillei* (TU - H. Milne Edwards, 1852) from Kenya; and *Tubuca urvillei* (TU - H. Milne Edwards, 1852), *Paraleptuca chlorophthalmus* (PC - H. Milne Edwards, 1837), and *Austruca occidentalis* (AO - Naderloo, Schubart & H.-T. Shih, 2016) from South Africa. The species were identified by using the *Atlas of crabs of the Persian Gulf* [59], the systematics of the indo-west Pacific broad-fronted fiddler crabs [60], and we double-checked the validity of the name in WORMS database [61] accessed the 3rd September 2022. The mangrove stands of Farasan Island, Saudi Arabia (KSA), consist of a fringing mangrove mainly composed of *Avicennia marina* trees. The average high temperature in summer reaches 35–37°C, with no precipitation. In the winter, average high temperatures are around 27–31°C, with precipitation up to 200 mm between November and April (data from Saudi Regional Climate Center). The tidal range is around max 1 m. However, the sea level also varies with season. In summer, the sea level drops and part of the mangrove remain exposed with no tidal influence, while in winter the sea level rises again and the mangrove is entirely flooded [62, 63]. The mangrove forest of Gazi Bay, Kenya (KY), extends up to 3.3 km across and surrounds the northern shores of the bay with an area of 6.61 km^2^ [64]. The mangrove receives low freshwater and sediment inputs. The tidal range is approximately 1.4 m during neap and 4 m during spring tides, generating significant flow across the bay. Water in-welling occurs from the large *Thalassodendron* seagrass beds, lying southwards and seawards of the mangroves, with significant retention of ebb currents by the mangroves themselves [65]. The climate on the Kenyan coast is typically monsoonal, with a rainy and wet season from March to September (southeast monsoons) and dry from October to March (northeast monsoons); rain however mainly falls between March and May. The total annual precipitation fluctuates between 1000 and 1600 mm. Air temperatures are high, with a mean of 27–28°C and little seasonal variation; relative humidity is around 95% [65]. The Mngazana estuary mangroves are situated in the Eastern Cape, South Africa (RSA), on the southeast coast. It is the third largest mangrove area in the country and covers approximately 118 ha [66]. The river flows through 275 km^2^ of catchment for 150 km before discharging into the Indian Ocean. The permanently open estuary is approximately 5.3 km long and hosts two river tributaries that support the main mangrove stands. Rainfall occurs throughout the year with more summer (November–January, 115.6 ± 3.4 mm) than winter rainfall (46.6 ± 3.1 mm) [66]. Annual minimum temperature ranges from 10.5 to 22.4°C and maximum temperature between 18.7 and 28.2°C. The mangrove forest in the Mngazana Estuary is one of the southernmost in the world.

From each site, 20 specimens per species (10 for molecular analysis and 10 for microscopy analysis) were collected (Fig. 1a); only adult male specimens with an average carapace width (± SE) of 2.5 cm ± 0.5 cm were selected. For comparison, 5 specimens of the swimming crab *T. crenata* were collected from Red Sea mangroves (KSA). Seven sediment cores measuring 5 cm in diameter by 5 cm deep were randomly taken in the area where the crabs were collected from each sampling site (KSA, KY and RSA). Seawater samples were also collected from the Red Sea mangroves (KSA). The crab gills were dissected under sterile conditions and preserved in RNA Later at 4°C, the sediment samples were kept frozen during transportation, and the seawater (5 L) samples were filtered through 0.2-μm sterile polyethersulfone (PES) Sterivex filters (Millipore) using a peristaltic pump (Millipore) and stored at −20°C until DNA extraction.

Gills were washed three times with sterile 0.9% physiological saline solution under sterile conditions before DNA extraction, as previously reported [67]. For sediment samples, DNA was extracted from 0.5 g of sediment using a PowerSoil DNA Isolation kit (MOBIO Laboratories, Carlsbad, CA, USA) as described in [68]. The total DNA from seawater was extracted following a modified version of the Phenol-Chloroform protocol described by Michoud and colleagues using lysis buffer, lysozyme solution and SDS-PK solution [69]. All the extracted DNA was visualised by ethidium bromide staining in agarose gel (0.8% TAE w/v) with electrophoresis and quantified using Qubit™ fluorometric quantification (Thermo Fisher, USA). The high-quality DNA was then used as template for shotgun metagenomic sequencing (see below) and 16S rRNA gene amplicon sequencing using the bacterial 341f and 785r primer pairs amplifying the V4–V5 hypervariable region as described by [70]. PCR products were purified using ExoSAP-IT™ (Thermo Fisher) and pooled at equimolar concentration before sequencing in the Bioscience Core Lab (BCL, KAUST) using Illumina MiSeq technology. Sequencing was performed using 300 bp paired-end sequence run libraries [71]. A total of 3,518,780 paired-end reads were obtained with lengths ranging from 240 to 285 bp after demultiplexing, with a mean of 450 bp per sample. Raw sequence reads have been deposited in the Short Reads Archive under BioProject ID numbers PRJNA294999 and PRJNA482213.

### Fiddler crab gills microscopy analysis

Gills from ten individual males per species were dissected and prepared for scanning (SEM) and transmission (TEM) electron microscopy. For SEM, entire gills were rinsed in phosphate buffer (PB) solution (0.1 M pH 7.2), then fixed with 2.5% glutaraldehyde in PB for 2 h at room temperature. Samples were subsequently rinsed with PB and post-fixed with 1% osmium tetroxide in the same buffer for 1 h. Specimens were then dehydrated by washes with increasing ethanol concentrations (50%, 75%, 95%, absolute) at room temperature. After dehydration, samples were critical point dried. Finally, the material was mounted on aluminium stubs, sputter coated with gold using a Balzers Med 010 and then examined with a Quanta 600 SEM, operating at 10 KV. For TEM, the gill sections (80 nm) were examined under a Zeiss EM900 transmission electron microscope, as previously described [72].

Fluorescent in situ hybridisation (FISH) was performed on three individual males of *A. albimana* to observe the distribution of the bacterial assemblage detected by Illumina sequencing (see above). The samples were fixed within 3 h of collection in 4% paraformaldehyde/phosphate-buffered saline (PBS, 3:1 vol:vol) for 12 h at 4°C, washed three times in ice-cold PBS and then stored at −20°C in 1:1 PBS/96% ethanol as described in [73]. An equimolar mixture of Cy3-labelled EUB338, EUB338II and EUB338III probes [74] was used for the detection of all bacteria, while for detecting specific bacterial phyla, we used a combination of probes as described in Supplementary Table S2. All hybridisations were performed at 40°C for 1.5–2.5 h following the protocols described by Cardinale and colleagues [75]. Formamide concentrations and other properties of the FISH probes are described in Supplementary Table S2. Washing steps with appropriate washing buffer matching the formamide concentration were carried out at 42°C for 10 min. A non-specific probe- or fluorochrome binding with sample tissues was checked by hybridisation of gill subsamples with an equimolar mixture of Cy3-, Cy5- and FITC-labelled NONEUB probes [76]. Stained samples were immediately dried with compressed air, mounted with Citifluor anti-fading medium (AF1; Electron Microscopy Science) and viewed within 24 h under a Leica TCS SP5 confocal laser scanning microscope equipped with argon and helium/neon lasers. For each field of view, an appropriate number of optical slices were acquired with a Z-step of 0.15−0.5 μm (“confocal stacks”); a minimum of 10 stacks were acquired for each gill. Confocal stacks were assembled for 3D reconstruction using the Imaris software (version 7.6.4; Bitplane, Zurich, Switzerland).

### 16S rRNA gene amplicon sequencing and data processing

The 16S rRNA gene amplicon sequences were pre-processed and analysed using UPARSE v8 [77] and QIIME v1.8 [78] software. Briefly, the paired-end reads for each sample were assembled using a minimum overlap of 50 nucleotides and a maximum of one mismatch within the region using the fastq-join algorithm (https://code.google.com/p/ea-utils/wiki/FastqJoin). The overlapped reads were quality filtered by trimming primer sequences and removing low-quality sequences. This file was imported into UPARSE, where operational taxonomic units (OTUs) of 97% sequence similarity were formed and chimaeras removed using de novo and reference-based detection. For reference chimaera detection, the “Gold” database containing the chimaera-checked reference database in the Broad Microbiome Utilities (http://microbiomeutil.sourceforge.net/) was used. Taxonomy was assigned to the representative sequences of the OTUs in QIIME using UClust [79] and searched against the latest version of the SILVA database (138 Version). Rarefactions were assessed, and all samples had an estimated coverage index of more than 97% (see Supplementary Figure S1). Finally, an OTU table (i.e. a sample x OTU count matrix with a tab containing the taxonomic affiliation of each OTU) was created. The OTU table and the phylogenetic tree were calculated with FastTree2 [80], using default parameters and the PyNast-aligned representative sequences as input. The OTU table and the phylogenetic tree were used as inputs for the subsequent analyses of alpha- and beta-diversity (weighted and unweighted UniFrac distances).

The Sloan’s neutral community model (SNCM) [81] was used to test whether the gill microbiome of each crab species was randomly recruited from the sediment at the respective site, and whether the microbiome in sediments from different sites were homogeneous. According to the model, in a neutrally assembled metacommunity governed by dispersal, the frequency of observing a taxon as a function of its mean relative abundance is described by a beta distribution. In other words, the model assumes that if a metacommunity is governed only by dispersal and random demographic processes (recruits and deaths), then an organism that is abundant at a given site should be frequently observed in the whole metacommunity. Thus, the model can be applied to test whether a metacommunity is neutrally assembled (or well-homogenised) or whether the assembly of a metacommunity is governed by dispersal from a “source” community. To fit the SNCM to the 16S rRNA gene amplicon data and to estimate the model’s goodness-of-fit and Akaike Information Criterion, we used the R code “sncm.fit_function.R” by [81].

The recently developed “phyloscore” framework [82] was used to detect bacterial phylogenetic clades with distinct phylogenetic turnover than that expected by chance in the crab gills microbiome. Lower-than-expected turnover is characteristic of clades having high niche occupancy across all the examined samples; in our case, this concerns crab gill samples across all sites. Similarly, higher-than-expected turnover is characteristic of clades having low niche occupancy, i.e. being present in a specific subset of samples. Briefly, the method consists of the following two steps: (1) The “phyloscore” is calculated for a given pair of communities, *j*,* k*, and for each OTU, *i*, that is present in one but not both communities. The phyloscore is a *z*-score quantifying how different the OTU’s nearest phylogenetic distance (i.e. nearest taxon distance - NTD) is to a null expectation in which species are randomly drawn to be present in the community in which OTU *i* is absent; (2) the total phyloscore for each OTU is then calculated as the sum of its phyloscores across all community pairs, and phylofactorisation is used to identify monophyletic clades of OTUs with significantly different total phyloscores compared to the complement set of OTUs and to extract the consensus taxonomic classification of the OTUs within [83]. We used the β mean nearest taxon distance (β-MNTD) to assess the phylogenetic β-diversity between the microbiomes of the gills among all the crabs. First, we constructed a phylogenetic tree (as described above) from the representative sequences of all the OTUs present in the crab gills. Then, we calculated the phylogenetic distances among all crab gill OTUs based on this tree and using the *cophenetic* function in R [84]. Finally, we used the resulting distance matrix to calculate the β-MNTD among all gill microbiomes with the *comdistnt* function in the “picante” package in R [85]. We tested the effect of “Site” on β-MNTD using the whole dataset and the effect of “Species” for each site separately (since all crab species were not present in each site) using the CAP analysis in PRIMER v7.

### Metagenomic shotgun sequencing, assembly and gene annotation

Paired-end libraries (2 × 250 bp) were prepared using the Truseq Library Preparation Kit (Illumina) according to the manufacturer’s protocol and sequenced on the HiSeq 4000 (Illumina) instrument in the Bioscience Core Lab at KAUST. Raw data for all 26 metagenomic datasets are deposited in NCBI under BioProject PRJNA680446 and summarised in Supplementary Table S3. In total, we sequenced gill microbiomes (*n* = 18) from three fiddler crab species and adjacent metagenomes from sediment (*n* = 8) collected near the crab burrows, yielding a total of 350 gigabase pairs of raw data.

The raw data with paired-end reads were quality filtered and trimmed using Trimmomatic v0.32 [86] to remove adapter sequences and leading and trailing bases with a quality score below 20 and reads with an average of 20 per base quality over a 4-bp window. This pre-processing step also included a mapping-based step to remove reads from the internal phage standard PhiX using BBmap v37.44 [87] with default settings. At each step, sequence quality was assessed using FASTQC v0.11.8 [88]. The resulting high-quality paired-end reads, averaging (± SD) 8.4 ± 3.7 Gbp per sample (Supplementary Table S4), were independently assembled de novo with metaSPAdes v3.9.0 [89], employing the error-correction mode, preset metagenomic options, and a kmer range of 21 to 127. Contigs shorter than 500 bp were excluded from further processing prior to gene prediction using Prodigal v2.6.0 [90] with default settings but in metagenomic mode. The predicted protein-coding genes (with a length of ≥100 bp) that comprised the crab microbiome (*n* = 18) and sediment microbiome (*n* = 8) totalled 1,796,269 genes, corresponding to an average (± SD) of 69,087 ± 81,348 genes per sample (Supplementary Table S5).

To aid taxonomic assignment and quantification of gene family abundance across the different crab species, we generated a catalogue of 1,640,845 protein-coding genes by de-replicating the ~1.8 million redundant genes (≥100 bp) from the fiddler crab and the reference sediments (*n* = 26) using cd-hit v4.6 [91]. De-replication was achieved by clustering all protein-coding gene sequences (CDS) at a global identity threshold of 95 and 80% coverage for the shorter gene; essentially following the clustering approach of UniRef gene families. The 1.64 million nonredundant genes were annotated using DMAP (formerly INDIGO, [92]). Annotation included functional prediction based on several reference databases, including the KEGG Orthology (KO) database [93], where a blast score of 70 was applied, and taxonomic assignment using the lowest common ancestor (LCA) approach based on NCBI’s nomenclature. Overall, two-thirds of the catalogued genes (1,119,202 genes) were annotated with putative functions in the various reference databases: Uniprot (1,147,692), InterPro (887,995), KEGG (737,124), and COG (651,433). More than half of the genes with KO functional identifiers (554,439 genes) were assigned to prokaryotes (95% Bacteria; ~2% Archaea). For downstream analyses, we excluded all KOs assigned to eukaryotes (7,068; ~3%) or viruses (351; 0.1%) as the first putative taxonomic rank (Supplementary Figure S8), as well as all unassigned KOs.

The resulting crab gill microbiome gene catalogue of 554,439 putative prokaryotic gene sequences with KO entries was mapped against the error-corrected reads for each sample separately with bowtie2 [94] using the “--sensitive" and “--qc-filter” options in addition to the default settings. This procedure generated a table of normalised gene abundances based on reads per kilobase per million mapped reads (RPKM) metric, resulting in a common metric of relative abundance of genes that accounts for variations in sequencing depth.

Finally, we focused on a set of genes comprising enzymes involved in carbon, nitrogen, and sulphur metabolism including the following: *AMT*, ammonia transporter (K03320); *GDH*, glutamate dehydrogenase (K15371); *GS*, glutamine synthetase (K01915); *soxC*, sulfite oxidase (K00387); *SQR*, sulphide:quinone oxidoreductase (K17218); *psrA*, polysulfide reductase subunit A (K08352); and *cutL*, aerobic carbon monoxide dehydrogenase, large subunit (K03520). The normalised gene abundances (RPKM) were then integrated with the corresponding taxonomic assignments of each gene of interest in the samples. The number of unique functions and corresponding functional diversity encompassing gene families (KOs) between crab gill and sediment microbiomes was assessed based on 100,000 genes randomly selected from the crab gill microbiome gene catalogue (with KOs and assignment to prokaryotes). Alpha (richness and Shannon) and beta (Bray-Curtis dissimilarity) metrics and hierarchical clustering to assess the grouping of samples were then performed and visualised using the R packages “FactoMineR” [95] and “ggplot2” [96], respectively. Random forest classification (100 iterations) was performed to identify KOs that were significant predictors of microbiome functional type (gill vs. sediment microbiome) using the “randomForest” package [97]. Cross-validation of the accuracy of the predictive model with six iterations for a 60% subset of samples showed that sample types (gill vs. sediment) were correctly identified 85% of the time based on the KOs abundance data.

### RNA extraction, sequencing and gene expression analyses

Total RNA was extracted from gill tissue of *Cranuca inversa* collected in Red Sea coastal waters near KAUST. Four separate animals were analysed, followed by total RNA extraction from the gill tissue samples and cDNA synthesis. Briefly, following the protocol from Callegari and colleagues [98], we used the RNeasy Mini kit (Qiagen) on the single entire gill homogenised in 350 µl Buffer RLT using sterile plastic pestles and added approximately 500 µl of sterile acid-washed glass beads with 425–600-µm diameter (Sigma) for a vortex step with the maximum speed for 5 min, then we followed the manufacturer instructions. A blank of extraction was also included. DNase I digestion of the extracted total RNA was performed for all the samples following the manufacturer’s instruction of the RNeasy Mini kit (Qiagen). The concentration of the extracted total RNA was evaluated using the QubitTM RNA broad range (BR) kit (Invitrogen), whereas the eventual contamination of gDNA was checked using the QubitTM dsDNA high-sensitivity (HS) kit (Invitrogen).

Messenger RNA (mRNA) was enriched from the total RNA extracts by depleting ribosomal RNA using the RiboZero kit for bacteria. Sequencing libraries (2 × 150 bp) were then prepared using the Truseq Stranded mRNA Library Preparation Kit (Illumina) according to the manufacturer’s protocol and sequenced on the HiSeq 4000 (Illumina) instrument in the Bioscience Core Lab at KAUST. The raw data for all 8 metatranscriptomes are deposited in NCBI under BioProject PRJNA680446 and summarised in Supplementary Table S6.

Pre-processing of the sequenced metatranscriptomes followed the same bioinformatics protocol as described above for metagenomes, using Trimmomatic and FASTQC for quality control. Subsequently, the remaining ribosomal RNAs (16S, 23S, 18S, and 5S) in the quality-checked reads were removed using SortMeRNA v2.1b [99] before the resulting high-quality mRNA reads (totalling 2–11 Gbp; Supplementary Table S6) were assembled using Trinity v2.13.2 [100] and subsequent identification of protein-coding sequences within the assembled long transcripts (16 to 62.3 kbp; Supplementary Table S6) with TransDecoder (https://github.com/TransDecoder), as described in Ngugi and colleagues [101]. The resulting transcripts were dereplicated with cd-hit v4.5.4 [102] at a global identity of 95% over 80% the length of the shorter gene and annotated with DMAP (formerly INDIGO, [77]) as described previously. Because the majority of assembled transcripts originated were eukaryotic/host-derived (60 to 76%; Supplementary Table S6) and to facilitate gene expression analysis of prokaryotes in the gills, we mapped the high-quality metatranscriptomes devoid eukaryotic reads against the dereplicated transcript catalogue of 195,388 nonredundant coding genes using BBMap v38.90 (https://github.com/BioInfoTools/BBMap) with default settings except for the options “idfilter=0.95 tossbrokenreads ambiguous=toss rpkm=%.rpkm pairedonly=t mapper=bbmap”. The “eukaryotic-free” metatranscriptomes were generated by screening the assemblies de novo with Whokaryotes [103] for eukaryotic transcripts and using these long eukaryotic transcripts as baits to remove putative eukaryotic reads in the high-quality metatranscriptomes with BBMap using the default settings.

### Classification of 16S rRNA genes from the metagenomes

16S rRNA gene reads present in the metagenomes (both bacteria and archaea) were extracted using SortmeRNA v2.1b [99]. The resultant 16S rRNA gene paired reads (Supplementary Table S3) were merged using Pandaseq v2.11 [104] with the parameters “-F -t 0.32 -A pear” prior to classification with MOTHUR v1.42.1 [105] based on the implemented SILVA database and taxonomic nomenclature (August 2020 release).

### Statistical analysis

Differences in alpha diversity metrics among sampling locations and species were analysed using an analysis of variance (ANOVA). To visualise differences in microbial community structure based on species abundances, Bray-Curtis dissimilarity matrices previously Log transformed were generated from OTU tables and subsequently subjected to PCoA using the R package “phyloseq” [106]. Homogeneity of multivariate dispersions between locations and species was tested using the function “betadisper” in the R package “vegan” [107] and was not found significant (betadisper, F_9,63_=1.4892, *p*=0.0684). A generalised linear model for a multivariate dataset, using the R package “mvabund” [108], was used to test for differences among bacterial communities at different locations (factor country, levels KY, RSA, and KSA representing Kenya, South Africa and Kingdom of Saudi Arabia, respectively, fixed and orthogonal) and in different species (factor species, levels TU, AA, CI, PC, and AO representing *T. urvillei*, *A. albimana, C. inversa, P. chlorophthalmus*, and *A. occidentalis*, respectively, and Sediment, fixed and orthogonal) and their interaction. Venn diagrams were computed with the R package MicEco (available at: https://github.com/Russel88/MicEco/blob/master/README.me). We tested the same experimental design for alpha diversity measures such as species richness, Shannon, Simpson, and Evenness indexes. The data were checked for normality and homogeneity of variance prior to all statistical analyses. All analysis was carried out with R software [109]. To explore the OTU occurrences between samples, following this experimental design, we built a bipartite network analysis using the qiime script *make_bipartite_network.py* and visualised with gephi [110].

### Supplementary Information

**Additional file 1: Video S1.** 3D structure of a crab gill lamellae obtained from integration of z-stacks of a set of confocal microscopy images taken after fluorescent in situ hybridisation with eubacteria and high GC Gram-positive bacteria specific fluorescent DNA probes.

**Additional file 2: Method S1.** Quantification of ammonia concentration in crab gills. Male individuals of *Cranuca inversa* and *Thalamita crenata* were collected from the Ibn Sina Field Research Station mangrove at KAUST (KSA) and kept in dedicated aquaria with fresh sediment for *C. inversa* and filtered fresh seawater flushed with air to maintain an oxygen saturation of 98%, at 21°C, 1 atm (assessed through a Fibox4 logger, Presence, Regensburg, Germany). After 12 h of acclimation, 10 individuals of each species were sacrificed, and the left gills were extracted. Gills were weighed and soaked with 300 µL of sterile ultrapure water (Invitrogen, Waltham, USA). Samples were then manually homogenised with plastic pestles for 1.5 µL tubes and centrifuged for 5 min, 13000g. After centrifugation, the supernatants were collected to be centrifuged again for another 10 min at 13000g. The final supernatant was used to quantify ammonia concentration in the crab gills using the ammonia assay kit MAK310 (Merck, Darmstadt, Germany) following the manufacturer's instructions. Fluorescence readings were performed with a TECAN infinite 200 pro spectrophotometer (TECAN, Grödig, Austria) in 96-well clear bottom black polystyrene microplates (Corning, NY, USA). Results were calculated following manufacturers’ indications and normalised on the fresh weight of initial gill tissue. **Table S1.** Pairwise comparison of the bacterial beta-diversity among Sites and Species (including sediments). **Table S2.** List of FISH probes used in this study. **Table S3.** General statistics for metagenomes and assemblies of fiddler gill and burrow sediments microbiomes. **Table S4.** Summary of 16S rRNA gene sequences retrieved from individual metagenomes under study. **Table S5.** List of KEGG orthology (KO) further investigated in this study related to carbon, sulfur, and nitrogen metabolism as well as the detoxification of sulfur compounds and xenobiotics. **Table S6.** General information and statistics for metatranscriptomes and assemblies from the gill tissues of the fiddler crab *C. inversa* collected in the Red Sea, KAUST coastline mangroves. **Figure S1.** Rarefaction (A) and Goods’ coverage index (B) of the bacterial 16S rRNA gene amplicon sequencing dataset. **Figure S2.** Eulero-Venn Diagram that shows the shared OTUs (numbers represent the percentage weighted for the OTUs relative abundance) among sediment, seawater and crab gills, and Taxonomy of the overall samples (A) and considering only the samples from Red Sea (B). **Figure S3.** (A) Taxonomical composition of bacterial communities in *T. crenata* and *C. inversa* highlights the large presence of *Ilumatobacter* sp. in the semiterrestrial fiddler crab gills and its paucity in the aquatic crabs. Notably, the “other Actinobacteria” detected in *T. crenata* mainly belonged to *Propionibacteriaceae* and *Microtrichaceae*. (B) Quantification of ammonia concentration in the crab gills. Significantly different ammonia concentrations on the gill of the swimming crabs *Thalamita crenata* and the fiddler crabs *Cranuca inversa* (Mann-Whitney test, U=14, *p*<0.0052, *n*=10). **Figure S4.** Constrained analysis of principal coordinates of the phylogenetic distances (alpha PD) among the bacterial microbiomes across (A) sites and (B) crab species. **Figure S5.** Scanning electron microscope imaging of fiddler crabs gill studied where we can see the constant coverage of the bacterial layer in all the species investigated. **Figure S6.** Bright-field (a) and FISH negative controls (b) of fiddler crabs gill lamellae. **Figure S7.** Metagenomic protein-coding gene sequence space of crab and bulk sediment microbiomes. (a) Total counts of predicted protein-coding genes in individual samples. (b) Relative abundance of prokaryotic (bacteria and archaea) relative to eukaryotic genes in sequenced metagenomes. (c) Phylum-level taxonomic breakdown of bacterial genes indicates. Actinobacteria’s prevalence in crab samples. **Figure S8.** Abundance and taxonomic assignment of unamplified 16S rRNA gene sequences retrieved from crab and bulk sediment metagenomes. (a) Total counts of 16S rRNA genes in individual samples. (b) Relative abundance of prokaryotic (bacteria and archaea) 16S rRNA genes in sequenced metagenomes. (c) Phylum-level taxonomic breakdown of bacterial 16S rRNA genes indicates Actinobacteria’s prevalence in crab samples. **Figure S9.** Krona graph showing the taxonomic breakdown of the representative protein-coding gene catalogue predicted from all metagenomes. Figure S10. Total counts of predicted protein-coding genes and the corresponding domain. Additional information is provided in Table S5.

## Data Availability

The datasets analysed during the current study are available in the NCBI SRA repository under the BioProject ID numbers PRJNA294999, PRJNA482213 and PRJNA680446.

## Abbreviations

rRNA: Ribosomal ribonucleic acid
DNA: Deoxyribonucleic acid
TSA: Tryptone soy agar
ANOVA: Analysis of variance
PERMANOVA: Permutational multivariate analysis of variance
PRIMER: Plymouth routines in multivariate ecological research
SEM: Scanning electron microscope
TEM: Transmission electron microscope
FISH: Fluorescent in situ hybridisation
CLSM: Confocal laser scanning microscopy
PBS: Phosphate-buffered saline
EDTA: Ethylenediaminetetraacetic acid
PCR: Polymerase chain reaction
QIIME: Quantitative Insights Into Microbial Ecology
OTU: Operational taxonomic unit
BC: Bray-Curtis
PCoA: Principal coordinate analysis
GLM: Generalised multivariate linear model
PERMDISP: Permutational analysis of multivariate dispersions
TU: *T. urvillei*
AA: *A. albimana*
CI: *C. inversa*
PC: *P. chlorophthalmus*
AO: *A. occidentalis*
GCCM: Gene catalogue of the crab gill microbiome
KEGG: Kyoto Encyclopedia of Genes and Genomes
KO: Kegg Orthology
RPKM: Reads Per Kilobase per Million mapped reads
ATP: Adenosine triphosphate
NADPH: Nicotinamide adenine dinucleotide phosphate
amoA: Ammonia monooxygase subunit A
amoB: Ammonia monooxygase subunit B
amoC: Ammonia monooxygase subunit C
CO: Carbon monoxide
H_2_S: Hydrogen sulphide
NH_3_: Ammonia
NH_4_^+^: Ammonium
SNCM: Sloan’s neutral community model
β-MNTD: Beta Mean Nearest Taxon Distance
CDS: Protein-coding gene sequences
LCA: Lowest common ancestor
AMT: Ammonia transporter
GDH: Glutamate dehydrogenase
GS: Glutamine synthetase
soxC: Sulphite oxidase
SQR: Sulphide:quinone oxidoreductase
psrA: Polysulfide reductase subunit A
cutL: Aerobic carbon monoxide dehydrogenase, large subunit
RSA: Republic of South Africa
KY: Kenya
KSA: Saudi Arabia
