# Supplementary material for: Gill-associated bacteria are homogeneously selected in amphibious mangrove crabs to sustain host intertidal adaptation
Source: Microbiome. 2023 Aug 24;11:189. doi: 10.1186/s40168-023-01629-4 (PMC10463870; doi:10.1186/s40168-023-01629-4)

ADDITIONAL FILE for

Gill-associated bacteria are homogeneously selected in amphibious mangrove crabs to sustain host intertidal adaptation

Marco Fusi[1,2,*,†], David K. Ngugi[1,3,†], Ramona Marasco[1], Jenny Marie Booth[1], Massimiliano Cardinale[4,5], Luciano Sacchi[6], Emanuela Clementi[6], Xinyuan Yang[1], Elisa Garuglieri[1], Stilianos Fodelianakis[1], Grégoire Michoud[1] and Daniele Daffonchio[1,*]

[1]Red Sea Research Center, Biological and Environmental Sciences and Engineering Division, King Abdullah University of Science and Technology, Thuwal 23955-6900, Kingdom of Saudi Arabia.

[2]Present address: Centre for Conservation and Restoration Science, Edinburgh Napier University, Edinburgh, UK

[3]Leibniz Institute DSMZ – German Collection of Microorganisms and Cell Cultures, Inhoffenstrasse 7B, D-38124 Braunschweig, Germany.

[4]Institute of Applied Microbiology Research Center for BioSystems, Land Use, and Nutrition (IFZ) Justus-Liebig-University Giessen, D-35392 Giessen, Germany.

[5]Department of Biological and Environmental Sciences and Technologies, University of Salento, via Prov.le Lecce-Monteroni, I-73100, Lecce, Italy.

[6]Dipartimento di Biologia e Biotecnologie "L. Spallanzani", Università di Pavia, I-27100, Pavia, Italy.

[†] M.F and D.K.N contributed equally to this work

***Corresponding authors**: daniele.daffonchio@kaust.edu.sa; marco.fusi@kaust.edu.sa

Supplementary Method

Method S1. Quantification of ammonia concentration in crab gills. Male individuals of *Cranuca inversa* and *Thalamita crenata* were collected from the Ibn Sina Field Research Station mangrove at KAUST (KSA) and kept in dedicated aquaria with fresh sediment for *C. inversa* and filtered fresh seawater flushed with air to maintain an oxygen saturation of 98%, at 21°C, 1 atm (assessed through a Fibox4 logger, Presence, Regensburg, Germany). After 12 h of acclimation, 10 individuals of each species were sacrificed, and the left gills were extracted. Gills were weighed and soaked with 300 µL of sterile ultrapure water (Invitrogen, Waltham, USA). Samples were then manually homogenised with plastic pestles for 1.5 µL tubes and centrifuged for 5 min, 13000*g*. After centrifugation, the supernatants were collected to be centrifuged again for another 10 min at 13000*g*. The final supernatant was used to quantify ammonia concentration in the crab gills using the ammonia assay kit MAK310 (Merck, Darmstadt, Germany) following the manufacturer's instructions. Fluorescence readings were performed with a TECAN infinite 200 pro spectrophotometer (TECAN, Grödig, Austria) in 96-well clear bottom black polystyrene microplates (Corning, NY, USA). Results were calculated following manufacturers' indications and normalised on the fresh weight of initial gill tissue.

Supplementary Tables

Table S1. Pairwise comparison of the bacterial beta-diversity among Sites and Species (including sediments).

**Pairs**	**F-model**	**r-square**	**adjusted-*p***
KSA_albimana vs KSA_inversa	2.765	0.217	**0.043**
KSA_albimana vs KSA_urvillei	3.104	0.154	**0.003**
KSA_albimana vs KSA_sediment	12.800	0.561	**0.004**
KSA_albimana vs KY_sediment	10.787	0.519	**0.002**
KSA_albimana vs KY_urvillei	7.444	0.383	**0.001**
KSA_albimana vs ZA_sediment	12.437	0.580	**0.003**
KSA_albimana vs ZA_occidentalis	2.585	0.190	**0.017**
KSA_albimana vs ZA_chlorophthalmus	1.800	0.141	0.087
KSA_albimana vs ZA_urvillei	3.597	0.217	**0.002**
KSA_inversa vs KSA_urvillei	5.882	0.257	**0.001**
KSA_inversa vs KSA_sediment	21.168	0.679	**0.004**
KSA_inversa vs KY_sediment	16.879	0.628	**0.003**
KSA_inversa vs KY_urvillei	12.065	0.501	**0.001**
KSA_inversa vs ZA_sediment	21.919	0.709	**0.006**
KSA_inversa vs ZA_occidentalis	8.811	0.445	**0.001**
KSA_inversa vs ZA_chlorophthalmus	3.402	0.236	**0.003**
KSA_inversa vs ZA_urvillei	5.110	0.282	**0.001**
KSA_urvillei vs KSA_sediment	12.760	0.429	**0.001**
KSA_urvillei vs KY_sediment	11.027	0.393	**0.001**
KSA_urvillei vs KY_urvillei	5.333	0.219	**0.001**
KSA_urvillei vs ZA_sediment	11.354	0.415	**0.001**
KSA_urvillei vs ZA_occidentalis	2.820	0.135	**0.002**
KSA_urvillei vs ZA_chlorophthalmus	4.413	0.197	**0.001**
KSA_urvillei vs ZA_urvillei	3.751	0.158	**0.001**
KSA_sediment vs KY_sediment	12.165	0.549	**0.003**
KSA_sediment vs KY_urvillei	38.467	0.762	**0.002**
KSA_sediment vs ZA_sediment	14.351	0.615	**0.002**
KSA_sediment vs ZA_occidentalis	18.688	0.629	**0.001**
KSA_sediment vs ZA_chlorophthalmus	12.602	0.534	**0.001**
KSA_sediment vs ZA_urvillei	16.965	0.566	**0.001**
KY_sediment vs KY_urvillei	30.006	0.714	**0.001**
KY_sediment vs ZA_sediment	1.077	0.107	0.35
KY_sediment vs ZA_occidentalis	15.683	0.588	**0.003**
KY_sediment vs ZA_chlorophthalmus	10.705	0.493	**0.001**
KY_sediment vs ZA_urvillei	14.452	0.526	**0.001**
KY_urvillei vs ZA_sediment	42.899	0.796	**0.002**
KY_urvillei vs ZA_occidentalis	11.060	0.460	**0.001**
KY_urvillei vs ZA_chlorophthalmus	7.001	0.350	**0.001**
KY_urvillei vs ZA_urvillei	4.142	0.216	**0.006**
ZA_sediment vs ZA_occidentalis	18.649	0.651	**0.003**
ZA_sediment vs ZA_chlorophthalmus	11.903	0.543	**0.001**
ZA_sediment vs ZA_urvillei	15.972	0.571	**0.001**
ZA_occidentalis vs ZA_chlorophthalmus	4.825	0.287	**0.001**
ZA_occidentalis vs ZA_urvillei	5.588	0.285	**0.001**
ZA_chlorophthalmus vs ZA_urvillei	4.116	0.227	**0.001**

Table S2. List of FISH probes used in this study.

**Name**	**Sequence (5'→3')**	**Fluorescent**	**Target group**	**% FA (40°C)**	**Reference**
EUB338*	GCT GCC TCC CGT AGG AGT	Cy3	Most bacteria	10	Amann R. I., Binder B. J., Olson R. J., Chisholm S. W., Devereux R. and Stahl D. A. (1990). Appl. Environ. Microbiol. 56: 1919−1925.
EUB338II*	GCA GCC ACC CGT AGG TGT	Cy3	*Planctomycetales*	10	Daims H., Brühl A., Amann R., Schleifer K.-H. and Wagner M. (1999). Syst. Appl. Microbiol. 22: 434−444.
EUB338III*	GCT GCC ACC CGT AGG TGT	Cy3	*Verrucomicrobiales*	10	Daims H., Brühl A., Amann R., Schleifer K.-H. and Wagner M. (1999). Syst. Appl. Microbiol. 22: 434−444.
ALF968	GGT AAG GTT CTG CGC GTT	Cy5	*Alphaproteobacteria*	35	Neef A. (1997). Anwendung der in situ Einzelzell-Identifizierung von Bakterien zur Populationsanalyse in komplexen mikrobiellen Biozönosen. Doctoral thesis (Technische Universität München).
BET42a	GCC TTC CCA CTT CGT TT	FITC	*Betaproteobateria*	35	Manz W., Amann R., Ludwig W., Wagner M. and Schleifer K.-H. (1992). Syst. Appl. Microbiol.15: 593 - 600.
GAM42a	GCC TTC CCA CAT CGT TT	Competitor probe for BET42a	*Gammaproteobacteria*	35	Manz W., Amann R., Ludwig W., Wagner M. and Schleifer K.-H. (1992). Syst. Appl. Microbiol.15: 593 - 600.
HGC236	AAC AAG CTG ATA GGC CGC	Cy5	*Actinobacteria*	10 - 20	Erhart, R., D. Bradford, R. J. Seviour, R. Amann, and L. L. Blackall (1997). Syst. Appl. Microbiol. 20:310-318:
ILU470	GTAGTTGGCCGCACCTTCTT	FITC	*Acidimicrobiales*	10 – 20	This work
NONEUB-Cy5	ACT CCT ACG GGA GGC AGC	Cy5	/	***	Wallner G., Amann R. and Beisker W. (1993). Cytometry. 14: 136−143.
NONEUB-Cy3	ACT CCT ACG GGA GGC AGC	Cy3	/	***	Wallner G., Amann R. and Beisker W. (1993). Cytometry. 14: 136−143.
NONEUB-FITC	ACT CCT ACG GGA GGC AGC	FITC	/	***	Wallner G., Amann R. and Beisker W. (1993). Cytometry. 14: 136-143.

* Applied as an equimolar mix; *** the same stringency conditions of the FISH probe with the same fluorescent

Table S3. General statistics for metagenomes and assemblies of fiddler gill and burrow sediments microbiomes.

Table S4. Summary of 16S rRNA gene sequences retrieved from individual metagenomes under study.

Table S5. List of KEGG orthology (KO) further investigated in this study related to carbon, sulfur, and nitrogen metabolism as well as the detoxification of sulfur compounds and xenobiotics.

Table S6. General information and statistics for metatranscriptomes and assemblies from the gill tissues of the fiddler crab *C. inversa* collected in the Red Sea, KAUST coastline mangroves.

Supplementary Figures

Figure S1. Rarefaction (A) and Goods' coverage index (**B**) of the bacterial 16S rRNA gene amplicon sequencing dataset.

A

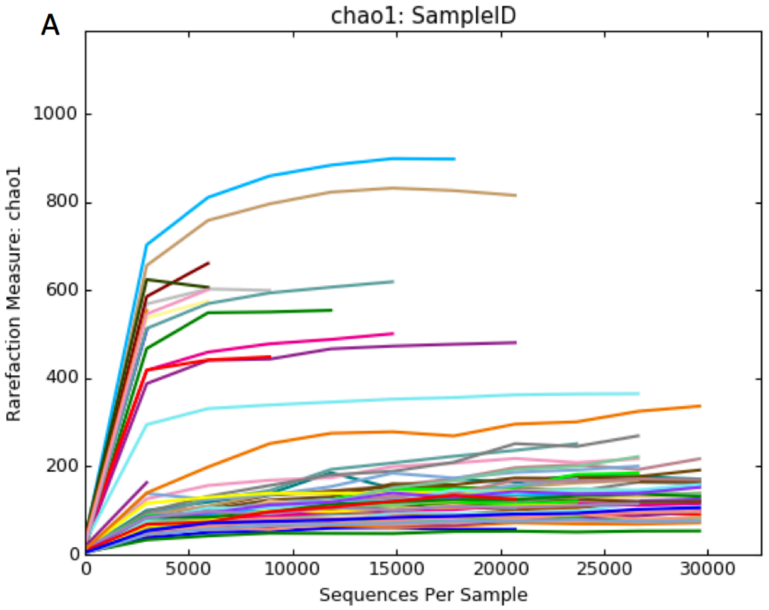

B

SampleID	Goods coverage	SampleID	Goods coverage
FARUA1	0.999994608	KY6	0.994948308
FARUA3	0.999440168	KYUU2	0.999354443
FARUA5	0.999851328	KYUU3	0.999256187
FARUA6	0.99976483	KYUU4	0.999157734
FARUA9	0.999777735	KYUU6	0.99953411
FARUA12	0.999648079	KYUU7	0.998893018
FARUA17	0.999370689	KYUU8	0.999016816
FARUI2	0.999612928	KYUU9	0.998555342
FARUI3	0.998851064	KYUU10	0.999382907
FARUI4	0.998688506	ZA1	0.987264151
FARUI5	0.999519031	ZA2	0.985878489
FARUI9	0.999670117	ZA3	0.995423649
FARUI10	0.999484967	ZA5	0.992638166
FARUU1	0.998187618	ZA6	0.99533022
FARUU2	0.999130948	ZAUA1	0.999391447
FARUU3	0.998923175	ZAUA3	0.999781372
FARUU3A	0.978070175	ZAUA4	0.999573204
FARUU4	0.998898326	ZAUA6	0.999010598
FARUU5	0.999122869	ZAUA7	0.999473833
FARUU6	0.999269419	ZAUA8	0.999663736
FARUU6A	0.999306218	ZAUA9	0.999409239
FARUU8	0.998716868	ZAUC1	0.998817656
FARUU9	0.999439695	ZAUC3	0.999566255
FARUU14	0.999341697	ZAUC9	0.999450412
FARUU19	0.999322418	ZAUC10	0.999728407
FARUU34	0.992413793	ZAUC11	0.990723562
KSA1	0.9806231	ZAUC12	0.9990341
KSA2	0.981242864	ZAUC13	0.999762963
KSA3	0.952734639	ZAUU1	0.997842813
KSA4	0.995811727	ZAUU2	0.997756142
KSA5	0.970710276	ZAUU3	0.998462104
KSA6	0.973694943	ZAUU4	0.997760024
KY1	0.992064692	ZAUU5	0.998595609
KY2	0.99794503	ZAUU6	0.999684357
KY3	0.999657112	ZAUU8	0.9998994
KY4	0.998951213	ZAUU10	0.999297788
KY5	0.986366898	ZAUU11	0.99931215

Figure S2. Eulero-Venn Diagram that shows the shared OTUs (numbers represent the percentage weighted for the OTUs relative abundance) among sediment, seawater and crab gills, and Taxonomy of the overall samples (**A**) and considering only the samples from Red Sea (**B**).

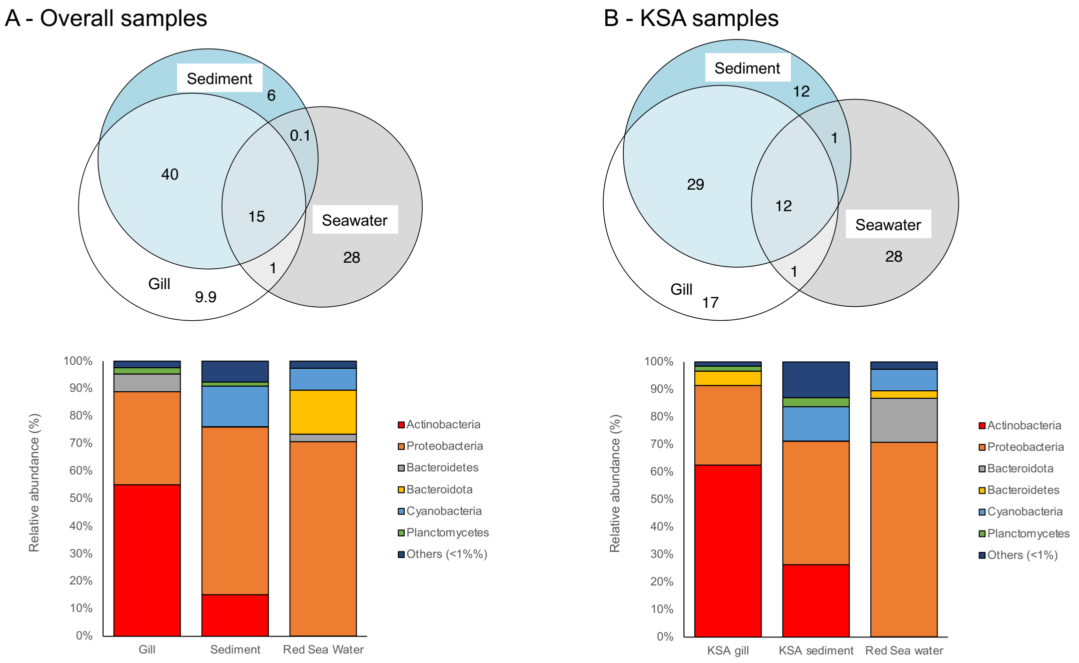

Figure S3. (**A**) Taxonomical composition of bacterial communities in *T. crenata* and *C. inversa* highlights the large presence of *Ilumatobacter* sp. in the semiterrestrial fiddler crab gills and its paucity in the aquatic crabs. Notably, the "other Actinobacteria" detected in *T. crenata* mainly belonged to *Propionibacteriaceae* and *Microtrichaceae*. (**B**) Quantification of ammonia concentration in the crab gills. Significantly different ammonia concentrations on the gill of the swimming crabs *Thalamita crenata* and the fiddler crabs *Cranuca inversa* (Mann-Whitney test, U=14, p<0.0052, n=10).

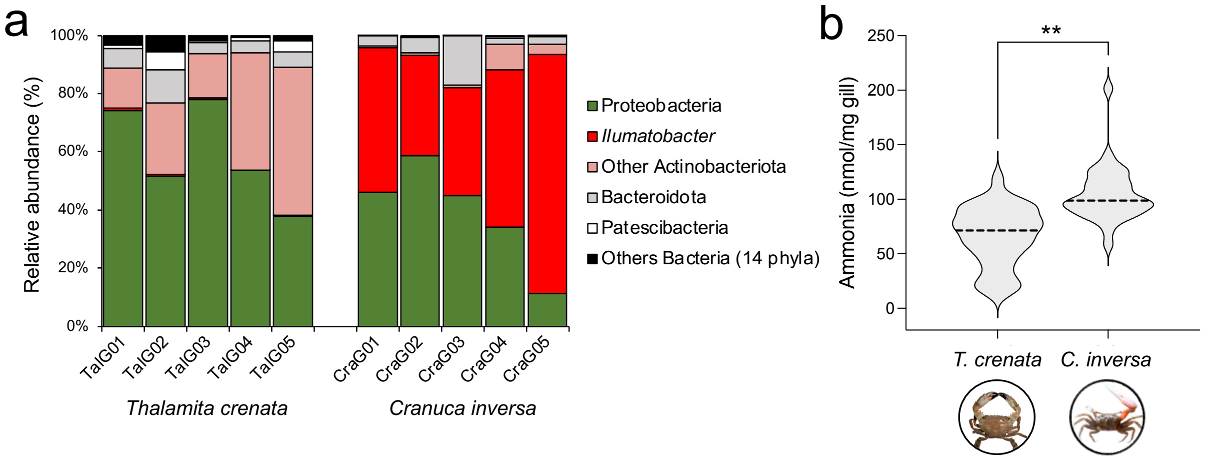

Figure S4. Constrained analysis of principal coordinates of the phylogenetic distances (alpha PD) among the bacterial microbiomes across (**A**) sites and (**B**) crab species.

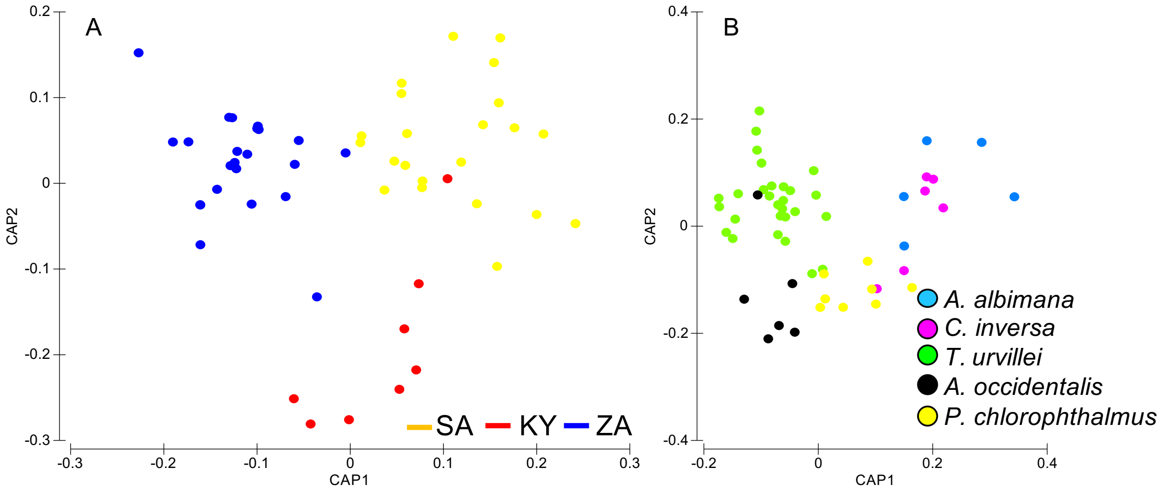

Figure S5. Scanning electron microscope imaging of fiddler crabs gill studied where we can see the constant coverage of the bacterial layer in all the species investigated.

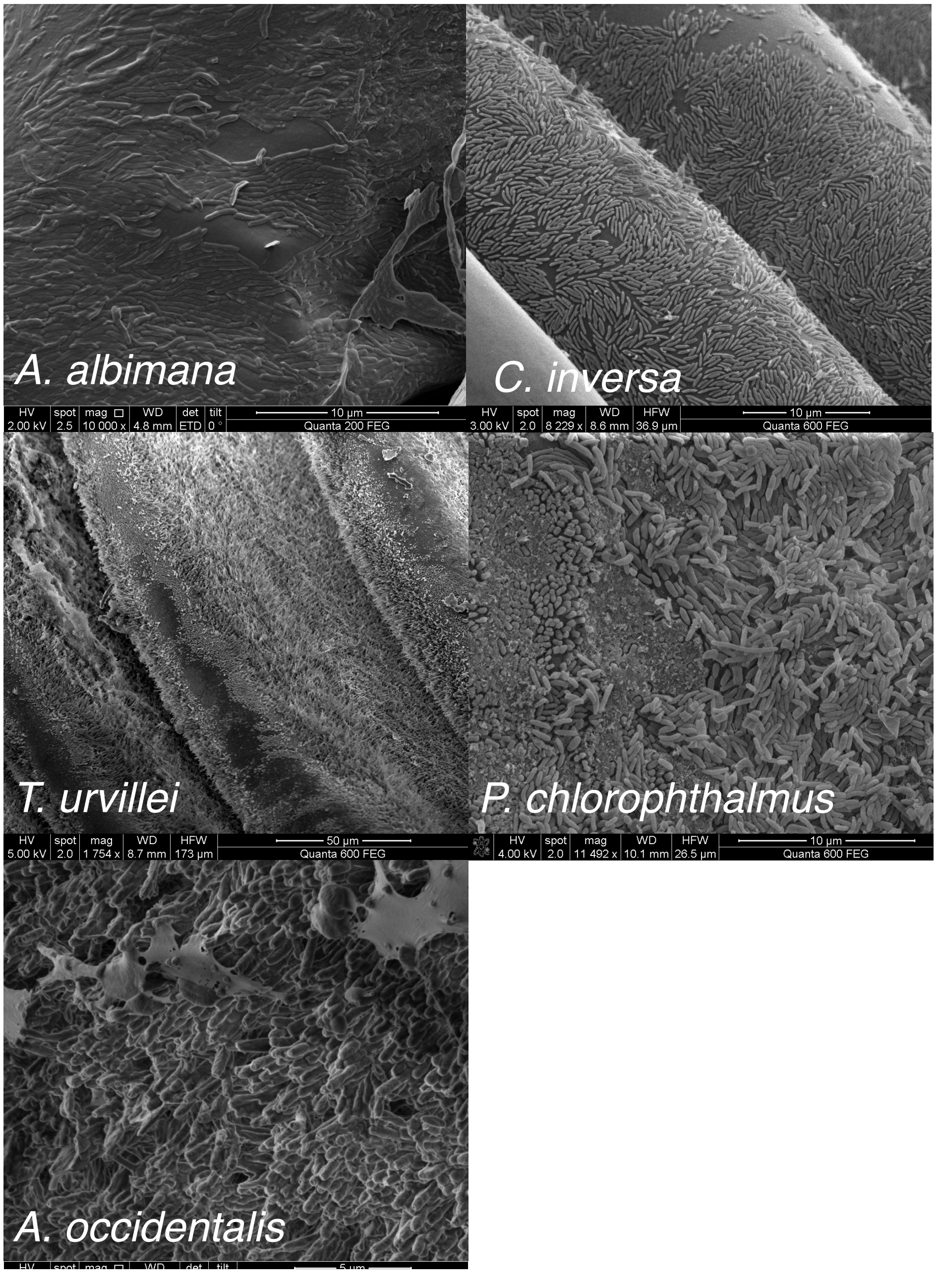

Figure S6. Bright-field (**a**) and FISH negative controls (**b**) of fiddler crabs gill lamellae.

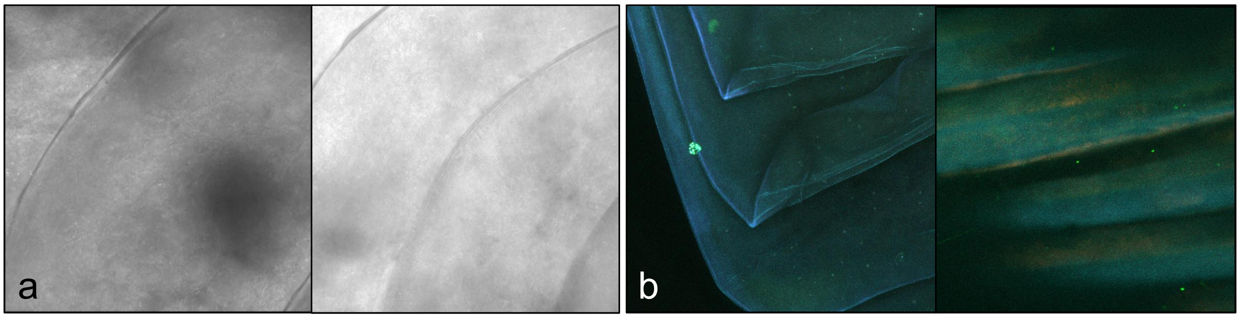

Figure S7. Metagenomic protein-coding gene sequence space of crab and bulk sediment microbiomes. (**a**) Total counts of predicted protein-coding genes in individual samples. (**b**) Relative abundance of prokaryotic (bacteria and archaea) relative to eukaryotic genes in sequenced metagenomes. (**c**) Phylum-level taxonomic breakdown of bacterial genes indicates Actinobacteria's prevalence in crab samples.

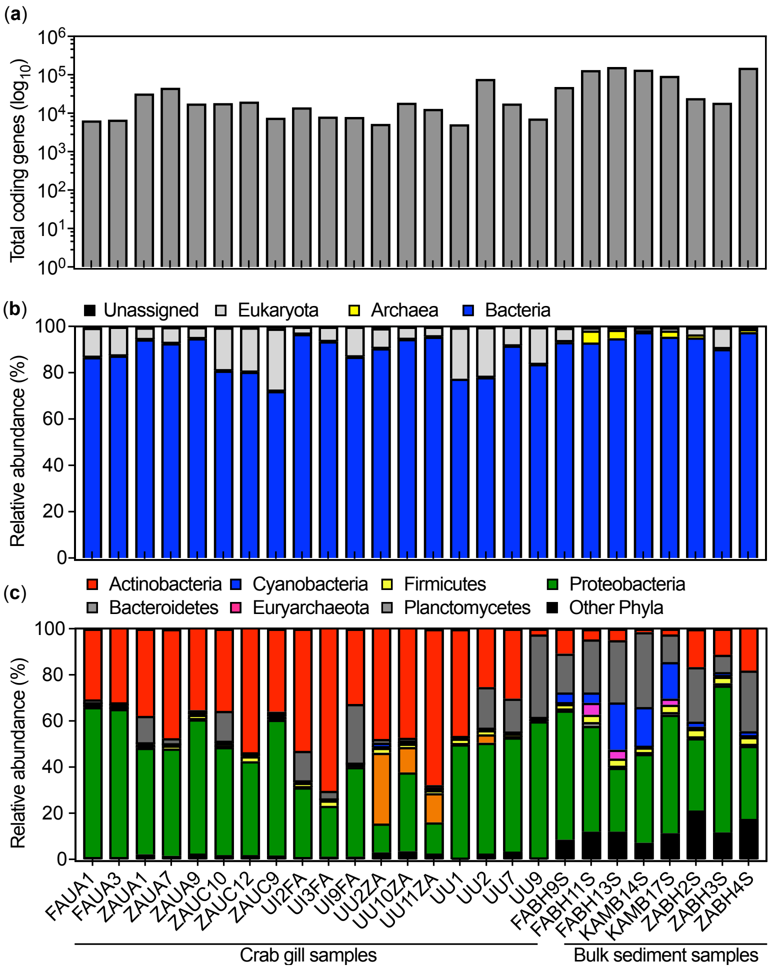

Figure S8. Abundance and taxonomic assignment of unamplified 16S rRNA gene sequences retrieved from crab and bulk sediment metagenomes. (a) Total counts of 16S rRNA genes in individual samples. (b) Relative abundance of prokaryotic (bacteria and archaea) 16S rRNA genes in sequenced metagenomes. (c) Phylum-level taxonomic breakdown of bacterial 16S rRNA genes indicates Actinobacteria's prevalence in crab samples.

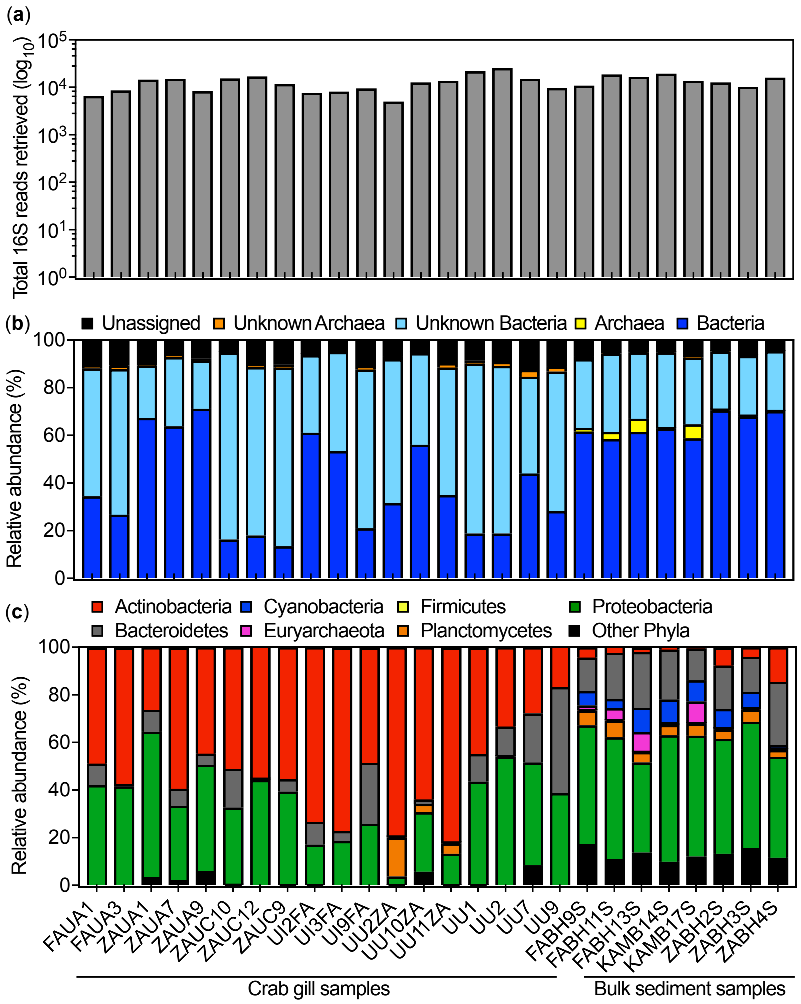

Figure S9. Krona graph showing the taxonomic breakdown of the representative protein-coding gene catalogue predicted from all metagenomes.

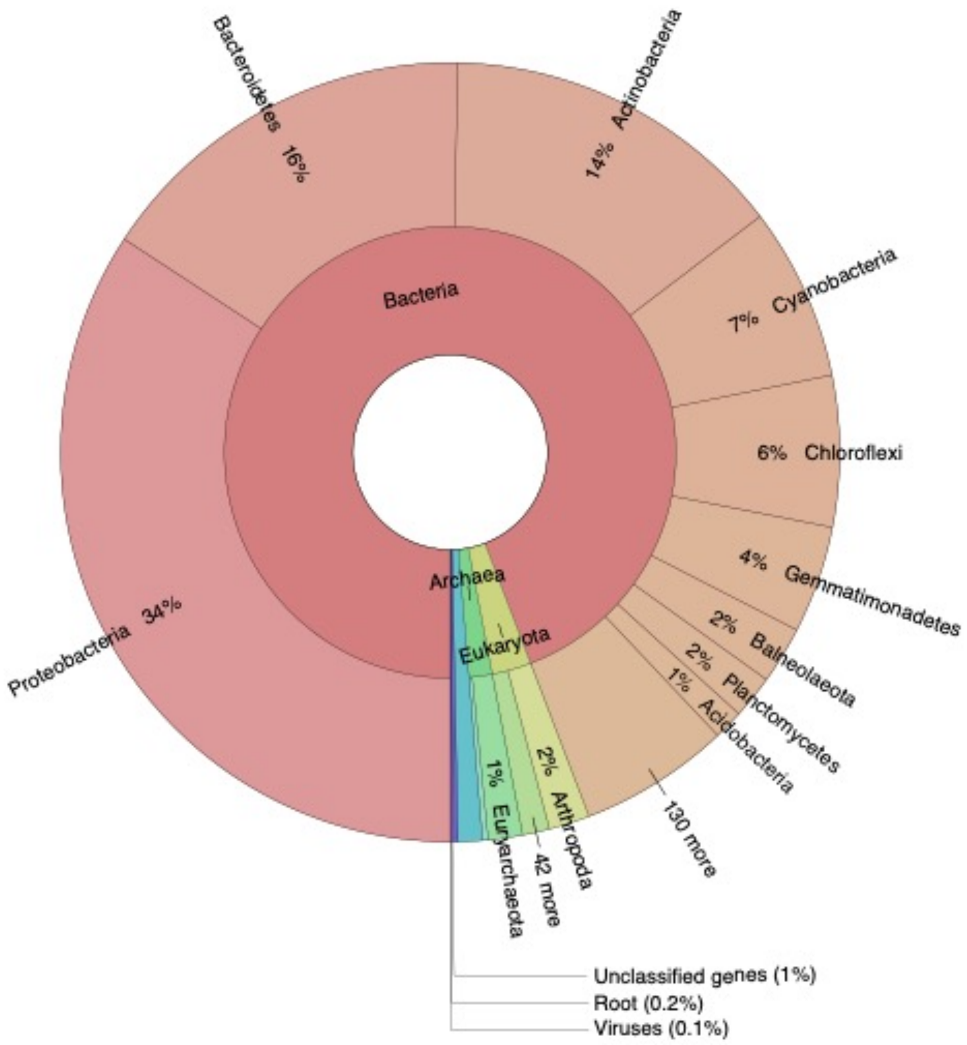

Figure S10. Total counts of predicted protein-coding genes and the corresponding domain. Additional information is provided in Table S5.

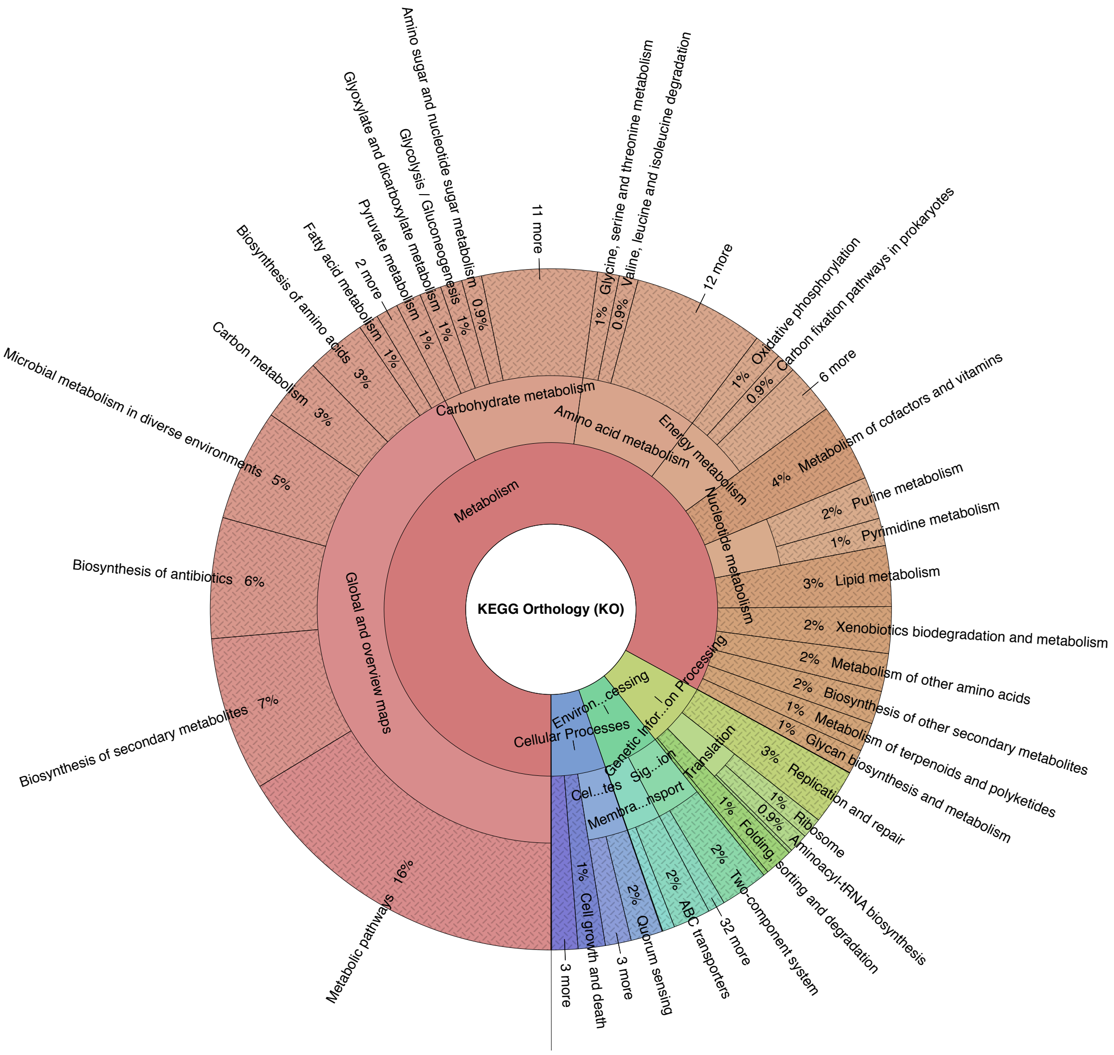

Supplement: Supplementary file 3 — Additional file 2: Method S1. Quantification of ammonia concentration in crab gills. Male individuals of Cranuca inversa and Thalamita crenata were collected from the Ibn Sina Field Research Station mangrove at KAUST (KSA) and kept in dedicated aquaria with fresh sediment for C. inversa and filtered fresh seawater flushed with air to maintain an oxygen saturation of 98%, at 21°C, 1 atm (assessed through a Fibox4 logger, Presence, Regensburg, Germany). After 12 h of acclimation, 10 individuals of each species were sacrificed, and the left gills were extracted. Gills were weighed and soaked with 300 µL of sterile ultrapure water (Invitrogen, Waltham, USA). Samples were then manually homogenised with plastic pestles for 1.5 µL tubes and centrifuged for 5 min, 13000g. After centrifugation, the supernatants were collected to be centrifuged again for another 10 min at 13000g. The final supernatant was used to quantify ammonia concentration in the crab gills using the ammonia assay kit MAK310 (Merck, Darmstadt, Germany) following the manufacturer's instructions. Fluorescence readings were performed with a TECAN infinite 200 pro spectrophotometer (TECAN, Grödig, Austria) in 96-well clear bottom black polystyrene microplates (Corning, NY, USA). Results were calculated following manufacturers’ indications and normalised on the fresh weight of initial gill tissue. Table S1. Pairwise comparison of the bacterial beta-diversity among Sites and Species (including sediments). Table S2. List of FISH probes used in this study. Table S3. General statistics for metagenomes and assemblies of fiddler gill and burrow sediments microbiomes. Table S4. Summary of 16S rRNA gene sequences retrieved from individual metagenomes under study. Table S5. List of KEGG orthology (KO) further investigated in this study related to carbon, sulfur, and nitrogen metabolism as well as the detoxification of sulfur compounds and xenobiotics. Table S6. General information and statistics for metatranscriptomes [file 40168_2023_1629_MOESM2_ESM.zip › Fusi_et_al_SI.pdf]
